# Van der Waals/MCT heterostructure enabled high-performance uncooled mid-infrared photodetectors via synergistic suppression of dark current and interfacial recombination

**DOI:** 10.1038/s41377-026-02418-y

**Published:** 2026-09-02

**Authors:** Xinlei Zhang, Tao Zhou, Yueying Cui, Ruizhi Li, Hao Wu, Yuwei Zhang, Rui Lin, Fang Yang, Kaiyang Liu, Jianfeng Wu, Dongyang Wan, Jialin Zhang, Junpeng Lu, Zhenhua Ni

**Affiliations:** 1 Key Laboratory of Quantum Materials and Devices of Ministry of Education, School of Physics, Southeast University, Nanjing, 211189 China; 2 Key Laboratory of State Manipulation and Advanced Materials in Provincial Universities, School of Physics and Technology, Nanjing Normal University, Nanjing, 210023 China; 3 School of Integrated Circuits, Southeast University, Nanjing, 210096 China; 4 School of Electronic Science and Engineering, Southeast University, Nanjing, 210096 China; 5 Advanced Ocean Institute, Southeast University, Nanjing, 210096 China

**Keywords:** Mid-infrared photonics, Photonic devices

## Abstract

Mid-wavelength infrared (MWIR) photodetection is crucial for applications such as night vision, remote sensing, spectral imaging and optical communication, yet its room-temperature operation faces a fundamental challenge of excessive dark current. While high-operating-temperature (HOT) HgCdTe (MCT) devices mitigate this issue through band structure engineering, their practical implementation is hindered by the intricate multilayer heteroepitaxy and lattice-mismatch-induced interfacial defects. To simultaneously resolve these bottlenecks, we develop a van der Waals (vdW) heterostructure strategy by integrating two-dimensional (2D) materials with MCT. The constructed MoS_2_/graphene/MCT vdW heterostructure synergistically addresses both the dark current and interfacial constraints. The p-n junction formed across the MoS_2_/graphene/MCT induces a strong built-in electric field and potential barrier, effectively suppressing dark current. Meanwhile, the interlayer graphene minimizes trap-assisted recombination and facilitates efficient photocarrier transport. Compared with MoS_2_/MCT and graphene/MCT heterojunctions, the MoS_2_/graphene/MCT vdW photodetector achieves an order-of-magnitude improvement in specific detectivity across visible to MWIR range. The optimized device architecture demonstrates a responsivity of ~0.325 A W^−1^ and a peak detectivity of ~8 × 10^10 ^cm Hz^1/2^ W^−1^ under room-temperature blackbody radiation, outperforming state-of-the-art uncooled MWIR photodetectors. This work provides a feasible strategy for designing high-performance uncooled MCT-based infrared photodetectors through 2D/MCT integration.

## Introduction

Room-temperature mid-wavelength infrared (MWIR) photodetectors are crucial across diverse domains including medical diagnostics^1^, autopilot systems^2^, security monitoring^3^, among others^4–6^. Among infrared-sensitive materials, HgCdTe (MCT) stands out for its tunable bandgap, superior infrared absorption, and long carrier lifetime, solidifying its dominance in high-performance infrared detection^7^. However, uncooled MCT photodetectors face a fundamental challenge: severe dark current induced by thermal excitation at ambient temperature, which degrades signal-to-noise ratios and limits room-temperature sensitivity^8^. While high-operating-temperature (HOT) MCT device architectures (e.g., N^+^/p/P^+^^9^, P^+^/N^−^/N^+^^10^, nBn^11^ structures) aim to suppress dark current through band engineering, their multilayer heterostructures suffer from interfacial defects caused by lattice mismatch and complex epitaxial growth process. These interfacial defects act as recombination centers, accelerating defect-assisted recombination of photogenerated carriers (which degrades carrier collection efficiency) while simultaneously contributing to dark current via Shockley-Read-Hall (SRH) recombination mechanisms^12^. These limitations highlight the urgent need for novel device architectures that simultaneously mitigate dark current and interfacial defects while maintaining fabrication simplicity.

Recent advances in van der Waals (vdW) integration of two-dimensional (2D) materials with bulk semiconductors offer a promising approach to address these challenges^13^. The atomically smooth and naturally passivated surfaces of 2D materials enable the construction of high-quality 2D materials/MCT (2D/MCT) vdW heterojunction, circumventing lattice mismatch issues^14–16^. Pioneering 2D/MCT studies have been demonstrated using graphene or black phosphorus (BP), achieving photovoltaic operation at zero bias with improved responsivity^17,18^. Nevertheless, these systems still exhibit elevated dark current density (~10^−4 ^A cm^−2^) limited by the gapless electronic structure of graphene and the narrow bandgap characteristic of BP. More critically, intrinsic surface defects in MCT remain unaddressed, acting as SRH recombination centers that suppress photogenerated carrier collection and elevate dark current^19,20^. To realize high-performance uncooled 2D/MCT-based photodetectors, a synergistic strategy is required: one that combines interface band alignment engineering with effective defect passivation to suppress both thermal excitation and interfacial recombination.

In this work, we propose a tri-layered MoS_2_/graphene/MCT vdW heterostructure designed to synergistically suppress dark current and interfacial recombination. The hybrid vdW heterostructure, comprising n-doped MoS_2_, p-doped graphene, and p-doped MCT, forms an effective p-n junction across the graphene-mediated interface. This band alignment establishes a built-in electric field across the heterostructure, enabling photovoltaic operation and suppressing dark current through majority-carrier blocking. Ultrafast carrier dynamics measurements elucidate the critical role of the interlayer graphene in mediating interfacial charge transfer and passivating defect states in MCT. This process effectively suppresses trap-assisted SRH recombination, thereby enhancing the quantum efficiency of the device. Compared with MoS_2_/MCT and graphene/MCT heterojunctions, this tri-layered design achieves an order-of-magnitude improvement in specific detectivity across visible to MWIR spectrum. The optimized device demonstrates an impressive responsivity of ~0.325 A W^−1^ and an ultra-high peak detectivity of ~8 × 10^10 ^cm Hz^1/2^ W^–1^ under blackbody radiation at room temperature, surpassing state-of-the-art uncooled MCT photodetectors. This innovative device architecture offers a feasible approach for designing next-generation MCT-based uncooled infrared photodetectors.

## Results

### Device structure design for suppressing dark current and interfacial recombination

**Fig. 1:**
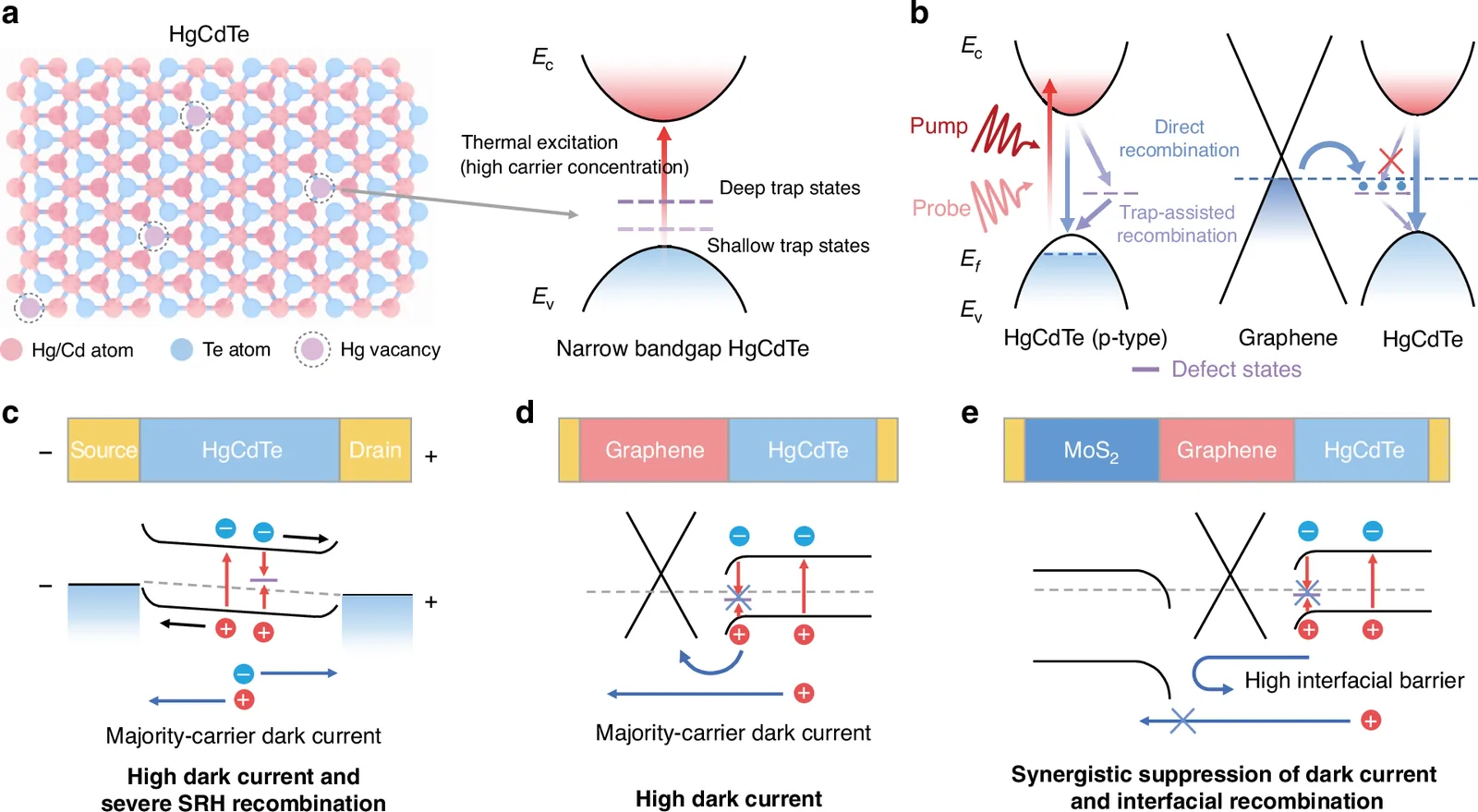
Schematic diagram of synergistic suppression of dark current and interfacial recombination of vdW/MCT heterostructure design. **a** Schematic diagram of HgCdTe crystal structure and Hg vacancy induced trap states. **b** Carrier recombination mechanism in MCT and suppression of interfacial recombination via graphene/MCT heterojunction. **c** Schematic diagram of severe SRH recombination and high dark current in MCT photoconductive detector. **d** Schematic diagram of high dark current issue in graphene/MCT vdW device. **e** Synergistic structure design of vdW/MCT device for suppressing dark current and interfacial recombination

Figure 1 outlines the design rationale for the device. The inherently weak Hg-Te bonds in as-grown p-type MCT thin films prepared by liquid phase epitaxy generate surface Hg vacancies, which introduce both shallow and deep trap states within the bandgap. The shallow trap states, which act as p-type acceptors, are located near the valence band maximum, while the deep trap states, typically situated at the mid-bandgap, serve as carrier recombination centers (Fig. 1a)^21^. The adverse trap states within the bandgap will participate in photoelectric conversion through carrier trapping and SRH recombination^22^, leading to shortened carrier lifetime, suppressed photoresponse, increased dark current, and severely degraded device performance^23^. To address the challenge of interfacial recombination, graphene was employed as a defect-passivation layer for its high electron density and large work function difference with MCT. As shown in Fig. 1b, electrons spontaneously transfer from graphene to MCT, filling recombination centers associated with vacancy defects and thereby effectively passivating interfacial trap states. Nevertheless, uncooled MCT photodetectors still suffer from high dark current due to strong thermal excitation at room temperature in the narrow bandgap materials. Figure 1c illustrates that the significant dark current in photoconductive MCT devices, dominated by majority carriers, elevates noise levels and impedes high-performance uncooled mid-wavelength infrared detection. While the graphene/MCT vdW heterostructure suppresses interfacial recombination, the high intrinsic carrier concentration of graphene and the low potential barrier still lead to a high dark current (Fig. 1d). To overcome these limitations, a MoS₂/graphene/MCT heterojunction architecture was designed to synergistically suppress interfacial recombination and block dark current through the introduction of a high interfacial barrier (Fig. 1e), offering a promising vdW-integration strategy for high-performance uncooled infrared photodetection.

The device configuration of the proposed uncooled MWIR van der Waals/MCT (vdW/MCT) heterostructure photodetector is shown in Supplementary Fig. S1, which features a vertically stacked MoS_2_/graphene/MCT tri-layered vdW structure. Polymethyl methacrylate (PMMA) is used as the dielectric layer for electric isolation, mitigating the impact of the temperature and numerous interface defects introduced during the dielectric layer deposition^18^. The p-doped MCT grown by liquid phase epitaxy serves as the infrared absorption layer, as confirmed by temperature-dependent Hall effect measurement and absorption spectra (Supplementary Fig. S2). The few-layer MoS_2_ and graphene, characterized as typical n-type and p-type behavior 2D materials (Supplementary Fig. S3), are assembled to form 2D/MCT vdW heterostructure, effectively blocking dark current and suppressing interfacial recombination. The fabrication process of MoS_2_/graphene/MCT vdW heterostructure device is shown in Supplementary Fig. S4. The cross-sectional transmission electron microscopy (TEM) image of vdW/MCT is depicted in Supplementary Fig. S5, revealing a flat and clean vdW interfaces without mismatch or damage. The atomic force microscopy (AFM) analyses (Supplementary Fig. S6) reveal that the thicknesses of the MoS_2_ and graphene sheets are ~7 nm and ~5 nm, respectively. The interfacial coupling effect and charge transfer of MoS_2_/graphene/MCT heterostructure are characterized via the Raman and photoluminescence (PL) spectra. The distinct observation of characteristic Raman peaks for MoS₂, graphene, and MCT in the tri-layered junction (Supplementary Fig. S7a) indicates the high quality of the transferred structure interface. Supplementary Fig. S7b displays the PL spectra collected from pristine MoS_2_, the MoS_2_/MCT heterojunction, and the tri-layered MoS_2_/graphene/MCT heterostructure, which exhibit expected PL quenching caused by interlayer charge transfer between the narrow and wide bandgap semiconductors. Compared with the MoS_2_/MCT heterostructure, the graphene-mediated 2D/MCT heterostructure exhibits more prominent PL quenching, implying more efficient charge separation enabled by the interlayer graphene. Scanning Kelvin probe microscopy (SKPM) was employed to elucidate the mechanism of interfacial recombination suppression and band alignment of MoS_2_/graphene/MCT heterostructure, as shown in Supplementary Fig. S8. For as-grown p-type MCT, the surface Hg vacancies introduce both shallow (∼24 meV above valence band) and deep (∼104 meV above valence band) trap states within the bandgap, according to calculation results (Supplementary Note S1). The significant work function difference (~176 meV) between the graphene and MCT facilitates spontaneous electron transfer from graphene to MCT, filling the empty trap states and suppressing trap-assisted recombination. Supplementary Fig. S8c, d illustrate the energy level alignment of tri-layer 2D/MCT heterostructure before and after contact, the type-II band alignment at the MoS_2_/MCT junction enables efficient separation of photogenerated carriers via the built-in electric field. Under infrared illumination, the photogenerated minority electrons within MCT are separated by the built-in electric field and transferred to the MoS_2_ layer with the assistance of the interlayer graphene, contributing considerable photocurrent. Meanwhile, the majority holes are blocked by the substantial interfacial barrier, ensuring low dark current.

### Interlayer charge transfer in graphene/MCT heterostructures

**Fig. 2:**
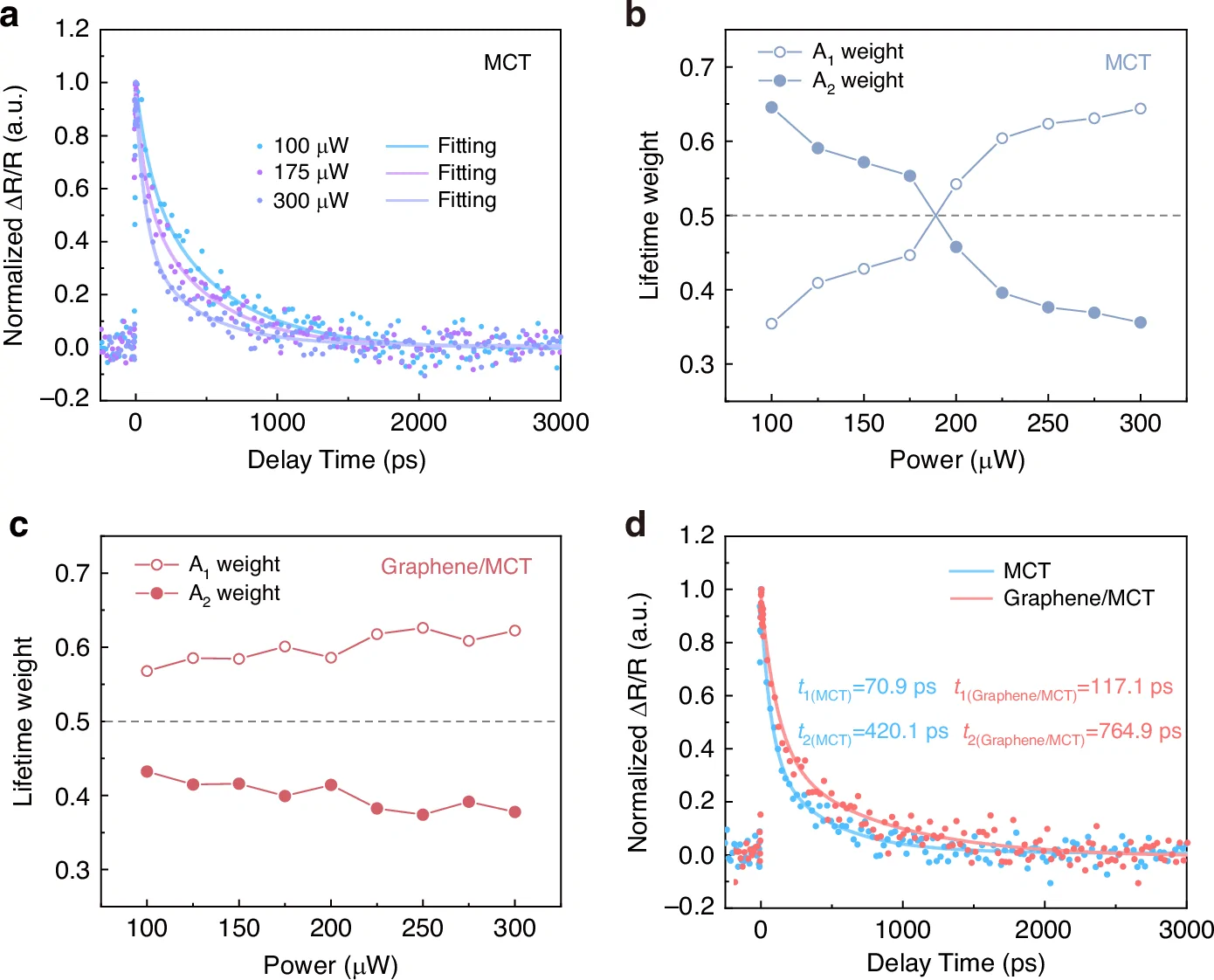
Transient reflectance spectra measurements of the graphene/MCT heterostructure and pristine MCT. **a** Power-dependent transient reflectance spectra (spots) with fitting curve (solid line) of MCT. **b** Power-dependent lifetime weights of pristine MCT thin film and **c** graphene/MCT heterostructure. **d** Carrier dynamics comparison of the graphene/MCT heterostructure and pristine MCT

To explore the interlayer charge transfer in the graphene/MCT heterostructure, transient reflectance spectra measured by pump-probe system were employed to investigate charge generation, transfer, and recombination processes. The two-color pump-probe system consists of an 800 nm laser pulse as the pump light and a 1100 nm laser pulse as the probe light. The optical microscope image of MoS_2_/graphene/MCT vdW heterostructure is shown in Supplementary Fig. S9. The power-dependent transient reflection decay profiles of photogenerated carriers of MCT are shown in Fig. 2a. The biexponential function, $\Delta R/{R}_{0}={\left(\Delta \frac{R}{{R}_{0}}\right)}_{\max }\left({A}_{1}{e}^{-t/{\tau }_{1}}+{A}_{2}{e}^{-t/{\tau }_{2}}+B\right)$, was employed to fit the carrier decay process. Here, ${\tau }_{1}$ and ${\tau }_{2}$ represent the short and long-time constants, corresponding to the fast and slow decay components, respectively. ${A}_{1}$ and ${A}_{2}$ denote the weight of these two components, while B is the weight of the long-lived residual signal. The decay processes of photo-generated carriers in the MCT sample can be well fitted with the biexponential function, indicating the presence of two distinct recombination mechanisms. The fast and slow decay time constants are extracted to be 50.0–105.4 ps and 405.2–606.3 ps, respectively. As shown in Fig. 2b, the relative weight of the fast and slow decay components in pristine MCT exhibits a clear power-dependent evolution. With increasing excitation power, the contribution of the slow decay process gradually decreases, while the fast decay component becomes increasingly dominant. This behavior can be attributed to the finite density of trap states within the MCT bandgap. At higher pump powers, the increased concentration of photogenerated carriers gradually fills these trap states, suppressing trap-assisted recombination and promoting direct interband recombination as the dominant decay path^24^. In contrast, the graphene/MCT heterostructure exhibits distinct power-dependent carrier decay and weight evolution, as shown in Supplementary Fig. S10a and Fig. 2c. The dominance of the fast decay process over the slow decay component across varying pump powers indicates suppressed trap-assisted recombination in the graphene/MCT heterostructure. This can be attributed to the high concentration of free electrons transferred from graphene to MCT, which can occupy the finite trap states induced by surface defects in the bandgap of MCT, thereby effectively passivating interfacial defect states. The interlayer charge transfer-induced passivation of defect states can be further corroborated by comparative ultraviolet photoelectron spectroscopy (UPS) measurements, which reveal modifications in the electronic structure and Fermi-level alignment at the graphene/MCT interface (Supplementary Fig. S11). The comparable decay dynamics and power-dependent weight distribution observed in both the MoS_2_/graphene/MCT heterostructure and the MoS_2_/MCT device indicate the feasibility of interlayer graphene in passivating interfacial trap states within the vdW/MCT heterostructure (Supplementary Fig. S10). The suppression of trap-assisted recombination significantly facilitates photo-generated carrier collection in the optoelectronic devices based on 2D/MCT vdW heterostructure.

Based on the above dynamic differences, we further compared the overall carrier lifetimes between the MCT and graphene/MCT, as shown in Fig. 2d. The carrier dynamics in the MCT exhibit a fast decay lifetime of 70.9 ps and a slow decay lifetime of 420.1 ps. In comparison, the graphene/MCT heterostructure shows significantly prolonged lifetimes, with the fast and slow decay components extending to 117.1 ps and 764.9 ps, respectively. The extended carrier lifetime observed in the graphene/MCT heterostructure is primarily attributed to recombination suppression induced by the built-in electric field at the graphene/MCT interface. This built-in field drives the spatial separation of photogenerated electrons and holes. As a result, photogenerated carriers are retained for a longer duration within the heterojunction, leading to a prolonged effective carrier lifetime compared to pristine MCT.

### Device optoelectronic properties under laser illumination

**Fig. 3:**
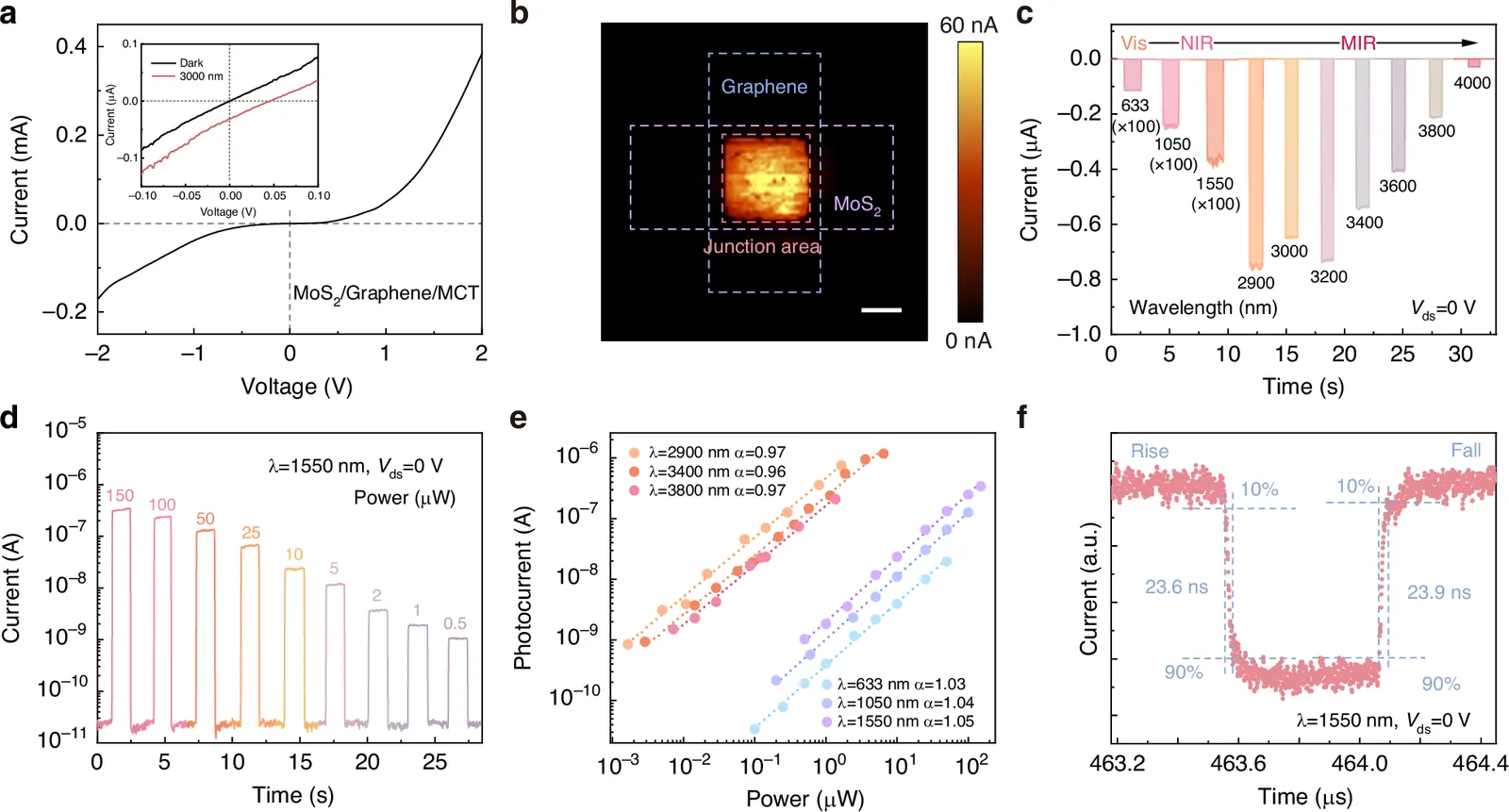
Optoelectronic properties of the MoS_2_/graphene/MCT vdW photodetector under laser illumination. **a** Output curve of the MoS_2_/graphene/MCT vdW heterostructure photodetector. Inset: measurement taken in the dark and under illumination. **b** Scanning photocurrent microscope mapping of the MoS_2_/graphene/MCT vdW heterostructure photodetector. The blue, purple, and red regions represent graphene, MoS_2_, and junction areas, respectively. The scale bar is 15 μm. **c** Wavelength-dependent temporal photoresponse from visible to MWIR spectra under zero bias. **d** Power-dependent temporal photovoltaic response under 1550 nm light illumination. **e** Power dependence of the photocurrent and corresponding fitting curve extracted at *V*_ds_ = 0 V for visible (633 nm), near-infrared (1050 nm, 1550 nm), and mid-infrared (2900 nm, 3400 nm, 3800 nm) bands. **f** Time-resolved photoresponse under 1550 nm at zero bias

The output characteristics of the MoS_2_/graphene/MCT heterojunction photodiode, measured at 300 K without light illumination, are shown in Fig. 3a and reveal the expected rectifying behavior of a diode formed with narrow-bandgap semiconductor thin film. The forward rectification behavior suggests the formation of a built-in electric field across the heterostructure. The inset of Fig. 3a displays the output characteristics under 3000 nm laser illumination and dark condition. The presence of photocarriers shifts the output curve downward, generating significant short-circuit current and open-circuit voltage, which confirms the photovoltaic operation of the proposed tri-layered 2D/MCT vdW photodetector. To clarify the photoresponse mechanism of the MoS_2_/graphene/MCT vdW photodetector, we performed scanning photocurrent microscopy mapping at 0 V bias under 633 nm laser illumination. As shown in Fig. 3b, the strongest photoresponse occurs in the overlapping area of the heterojunction, exhibiting exact spatial correlation with the optical image (Supplementary Fig. S12a). This indicates the formation of an effective heterojunction region between MoS_2_, graphene, and MCT. We then systematically investigated the photovoltaic response across the visible to MWIR range and measured the temporal photovoltaic response under various light powers. The wavelength-dependent temporal photovoltaic response with 2 μW incident light power from 633 nm to 4000 nm is shown in Fig. 3c, indicating broadband photodetection performance from visible to MWIR region. Figure 3d illustrates power-dependent photoresponse under 1550 nm incident light and temporal photoresponse under visible and near-infrared light are presented in Supplementary Fig. S12. The sharp current increase or decrease upon turning the incident light on or off indicates the sensitive photoresponse of the vdW heterostructure device, with the photocurrent $({I}_{{ph}}={I}_{{on}}-{I}_{{off}})$ increasing as the incident light power rises. Benefiting from the significant interfacial barrier for blocking the majority carrier transport from narrow bandgap layer^25^, the MoS_2_/graphene/MCT vdW heterostructure photodetector demonstrates a dark current of ~10 pA under zero bias and a light on-off ratio (*I*_on_/*I*_off_ ratio) exceeding 10^4^. The time-resolved photoresponse of the MoS_2_/graphene/MCT vdW photodetector under various MWIR illumination power densities (3.0 μm to 4.0 μm) is shown in Supplementary Fig. S13. The diminished photoresponse in the longer wavelength region is consistent with the optical absorption spectrum (Supplementary Fig. S14a), indicating the MWIR photoresponse orginates from the direct interband transitions. Figure 3e displays net photocurrent as a function of incident light power $({P}_{{in}})$ for visible, near infrared, and MWIR light, expressed by a power law: ${I}_{{ph}}\propto {P}_{{in}}^{\alpha }$, where power exponents $\alpha \,(0\le \alpha \le 1)$ reflects the involvement of trap states in the photoelectric conversion process^26^. The extracted α value ranges from 0.96 to 1.05. This suggests the high quality of the MoS_2_/graphene/MCT vdW heterostructure, as the presence of deep trap centers typically results in a sublinear relationship. The power-dependent photoresponsivity (*R*) under MWIR, calculated by $R={I}_{{ph}}/{P}_{{in}}$, is shown in Supplementary Fig. S14b. The device exhibits an average photoresponsivity exceeding 0.29 A W^−1^ across the 2900–3600 nm spectral range with a peak value ~ 0.64 A W^−1^ under 3200 nm illumination, while demonstrating negligible dependence on incident light power within the MWIR regime. The response speed of a photodetector is crucial as it determines the ability of the device to rapidly and accurately capture and process changing optical signals, which is essential for high-frequency applications, real-time imaging, and optical communication^27^. The response time of the device under 1550 nm laser illumination at 0 V bias was measured, showing a rise time (${\tau }_{r}$) of 23.6 ns and a fall time (${\tau }_{f}$) of 23.9 ns, corresponding to the photocurrent transition between 10% and 90% levels, as derived from the temporal response in Fig. 3f. Supplementary Fig. S15 shows the time-resolved response of tri-layered vdW/MCT device at a frequency of 5 MHz and its relative response versus switching frequency, revealing a −3 dB cutoff frequency of 59 MHz. Notably, under mid-wave infrared pulsed laser illumination, the device exhibits a comparable response speed on the same order of magnitude (~34 ns, Supplementary Fig. S16), demonstrating the consistency of the photoresponse across different spectral regimes. The vdW/MCT device exhibits excellent stability, maintaining a similar photoresponse after three months with negligible degradation (Supplementary Fig. S17).

### Optoelectrical characteristic comparison between 2D/MCT heterojunction and tri-layered 2D/MCT vdW structure

**Fig. 4:**
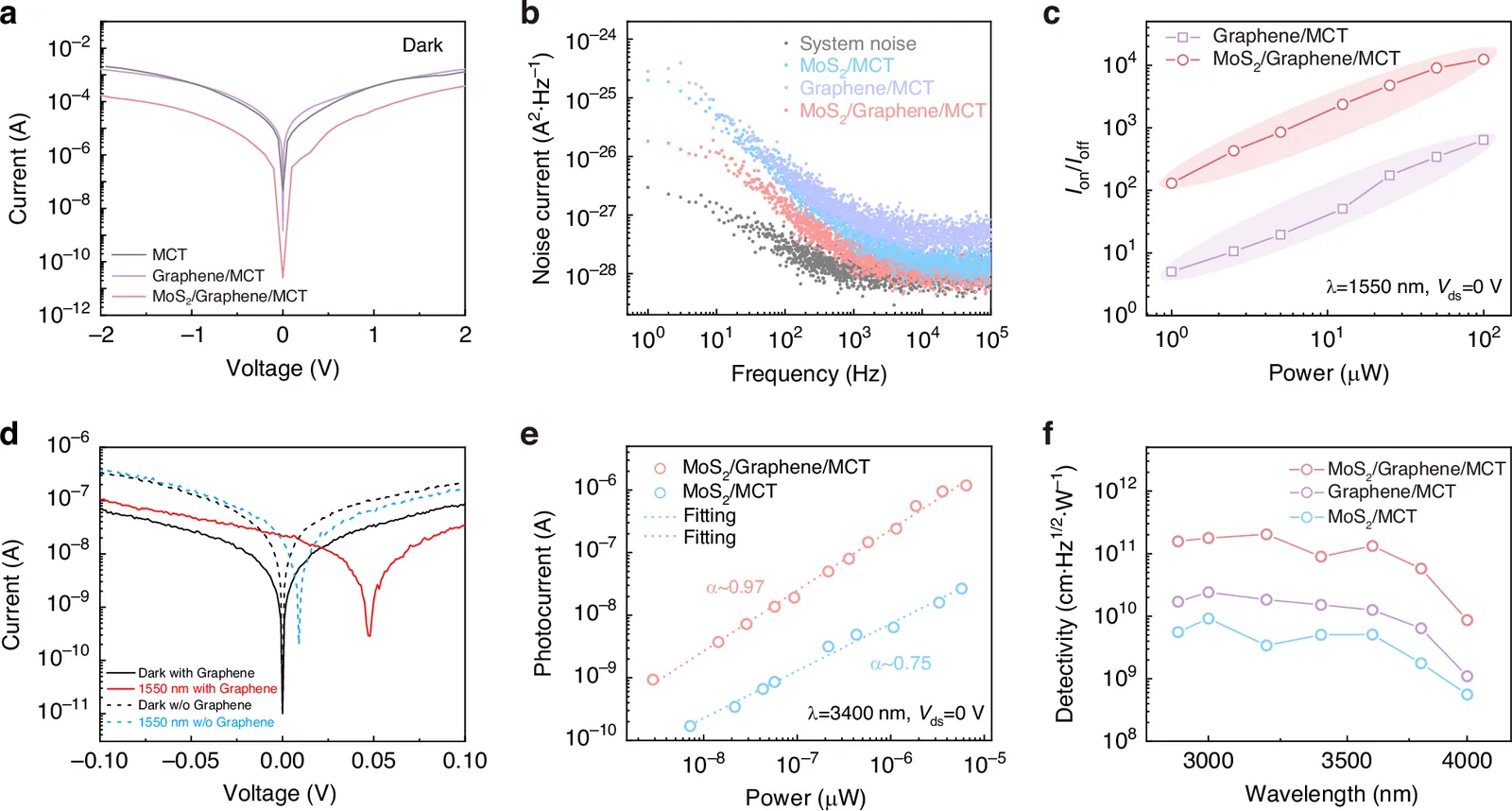
Optoelectrical characteristic comparison between 2D/MCT heterojunction and tri-layered vdW/MCT heterostructure. **a** Output curves comparison of the MCT device, the graphene/MCT vdW heterojunction photodetector, and the MoS_2_/graphene/MCT vdW photodetector. **b** Noise power density spectra comparison of the 2D/MCT vdW heterojunction and MoS_2_/graphene/MCT vdW structure. **c** *I*_on_*I*_off_ ratio comparison between the graphene/MCT heterojunction and MoS_2_/graphene/MCT vdW photodetectors. **d** Open-circuit voltage and comparison between MoS_2_/MCT heterojunction and MoS_2_/graphene/MCT vdW photodetector under 1550 nm light illumination. **e** Power-dependent photocurrent and fitting curves comparison between the MoS_2_/MCT heterojunction and MoS_2_/graphene/MCT vdW photodetector under 3400 nm light illumination. **f** Comparison of wavelength-dependent specific detectivity under MWIR region between the graphene/MCT heterojunction, MoS_2_/MCT heterojunction, and MoS_2_/graphene/MCT vdW photodetector

To understand the different roles of graphene and MoS_2_ in 2D/MCT vdW photodetectors and the superiority of proposed tri-layered 2D/MCT vdW structure, we conducted a comparative analysis of electrical and optoelectrical characteristics between 2D/MCT heterojunction and tri-layered vdW/MCT structure. Figure 4a displayed output curves of MCT device, graphene/MCT heterojunction, and MoS_2_/graphene/MCT vdW device. Photoconductive device based on MCT exhibits large dark current due to the high carrier concentration nature of narrow bandgap semiconductors. The construction of graphene/MCT heterojunction can remarkably reduce dark current at zero bias by introducing Schottky barrier. However, limited by the gapless nature of semimetal graphene and relatively small interfacial barrier, the graphene/MCT heterojunction still suffers from a high dark current^28^. Benefiting from the significant interfacial barrier between the MoS_2_, the proposed tri-layer 2D/MCT vdW structure can effectively reduce the dark current by more than an order of magnitude compared with graphene/MCT and the zero-bias resistance-area product (${R}_{0}A$) is enhanced nearly two orders of magnitude (Supplementary Fig. S18). The noise current of photodetector is correlated with dark current and trap state density, including 1/*f* noise, shot noise and thermal noise^29^. Figure 4b compared the noise power density spectra of the 2D/MCT vdW heterojunction and MoS_2_/graphene/MCT vdW photodetector without external bias voltage. Under low-frequency regime (*f* < 1 kHz), the noise power density spectra of 2D/MCT heterojunction and tri-layer 2D/MCT vdW photodetector both exhibit linear dependence with the frequency, which can be attributed to the 1/*f* noise^30^. Compared with MoS_2_/MCT vdW heterojunction, the tri-layered 2D/MCT vdW device exhibits lower noise current at low-frequency regime, suggesting a more prominent trap states associated-carrier generation-recombination process within depletion region in 2D/MCT vdW device without graphene interlayer^31^. At higher frequency, the noise power density spectra is independent of frequency and nearly maintain a constant value, indicating that shot noise and thermal noise dominate the noise current. The elevated dark current of graphene/MCT device leads to increased shot noise, resulting in a higher noise current beyond 1 kHz. From the measured noise power density spectra, we can calculate the overall noise current across the bandwidth of 10^5 ^Hz by ${I}_{n}=\sqrt{{\int }_{1}^{\Delta f}{S}_{n}(f){df}}$ ^32^. The calculated ${I}_{n}$ values are 7.4 × 10^−12 ^A, 4.4 × 10^−12 ^A, and 3.5 × 10^−12 ^A for graphene/MCT heterojunction, MoS_2_/MCT heterojunction, and MoS_2_/graphene/MCT vdW photodetector respectively. The lower noise current enables higher sensitivity and recognition ability of weak infrared signals. Figure 4c shows *I*_on_/*I*_off_ ratio comparison between the graphene/MCT heterojunction and MoS_2_/graphene/MCT vdW photodetector which is extracted from the temporal photoresponse under 1550 nm light illumination (Supplementary Fig. S19). The MoS_2_/graphene/MCT vdW photodetector demonstrated an order of magnitude higher *I*_on_/*I*_off_ ratio than graphene/MCT heterojunction from 1 μW to 100 μW, demonstrating excellent photoresponse and better weak infrared signal detection of the MoS_2_/graphene/MCT vdW photodetector.

The interlayer recombination suppression effect of graphene in the MoS_2_/graphene/MCT vdW heterostructure is further analyzed via open-circuit voltage $\left({V}_{{oc}}\right)$ measurements. Figure 4d compares the output characteristic curves of the MoS_2_/graphene/MCT vdW heterostructure device and MoS_2_/MCT vdW heterojunction device, with and without 1550 nm light illumination. The MoS_2_/graphene/MCT vdW heterostructure device shows an open-circuit voltage of 47 mV, in contrast to the much smaller values $\left({V}_{{oc}}\, \sim \,9\,{{\rm{mV}}}\right)$ for the MoS_2_/MCT device under 1550 nm laser power illumination. The enhanced ${V}_{{oc}}$ originates from the lower trap density within the depletion region, which reduces the nonradiative recombination of photogenerated carriers and improves the junction quality^33^. The ${V}_{{oc}}$ versus the incident light power density is plotted in Supplementary Fig. S20a, showing an enhancement in open circuit voltage with the increase of incident light power. Under steady-state open-circuit condition, the carrier densities in p–n junctions are determined by the balance between photogeneration and the interlayer recombination rate. The produced open-circuit voltage can be described with $\frac{d\left({V}_{{oc}}\right)}{d({{\mathrm{ln}}}_{{P}_{{opt}}})}=\frac{{2k}_{B}T}{\gamma q}$, where ${k}_{B}$ is the Boltzmann constant, T is the temperature, q is the elementary charge^34^. The factor γ denotes the recombination order, γ=1 indicates that recombination is dominated by monomolecular (SRH) process, while γ=2 indicates bimolecular (Langevin) process. The $\gamma \sim 1.73$ was extracted by fitting the experimental data (Supplementary Fig. S20b), indicating that the interlayer recombination is dominated by the Langevin process. The subordinate dominance of SRH recombination verifies that the interlayer graphene can effectively reduce interfacial trap states and thus repress interfacial trap-associated recombination. The observed sublinear photocurrent-power dependence in MoS_2_/MCT heterojunctions, combined with broadband photocurrent enhancement from visible to MWIR wavelengths in the tri-layered vdW/MCT architecture, indicates graphene-mediated effective interlayer recombination suppression and efficient interlayer charge transfer. (Fig. 4e and Supplementary Fig. S21a, b). Specific detectivity, as a key figure of merit for evaluating the sensitivity of the detector, is influenced by both responsivity and noise current effect, which can be calculated by formula ${D}^{* }={\left({A}_{D}B\right)}^{1/2}R/{i}_{n}$, where ${A}_{D}$ is the active area of detector and B is the bandwidth. As illustrated in Fig. 4f, the MoS_2_/graphene/MCT vdW photodetector exhibits a specific detectivity exceeding 5.0 × 10^10 ^cm Hz^1/2^ W^−1^ from 2900 nm to 3800 nm with a peak value of 2.0 × 10^11 ^cm Hz^1/2^ W^−1^ at 3200 nm laser illumination. Notably, the device demonstrated more than an order of magnitude improvement in specific detectivity compared with graphene/MCT heterojunction and MoS_2_/MCT heterojunction, underscoring the synergistic optimization enabled by the 2D/MCT vdW heterostructure architecture.

### Blackbody characterization of the MoS_2_/graphene/MCT vdW photodetector

**Fig. 5:**
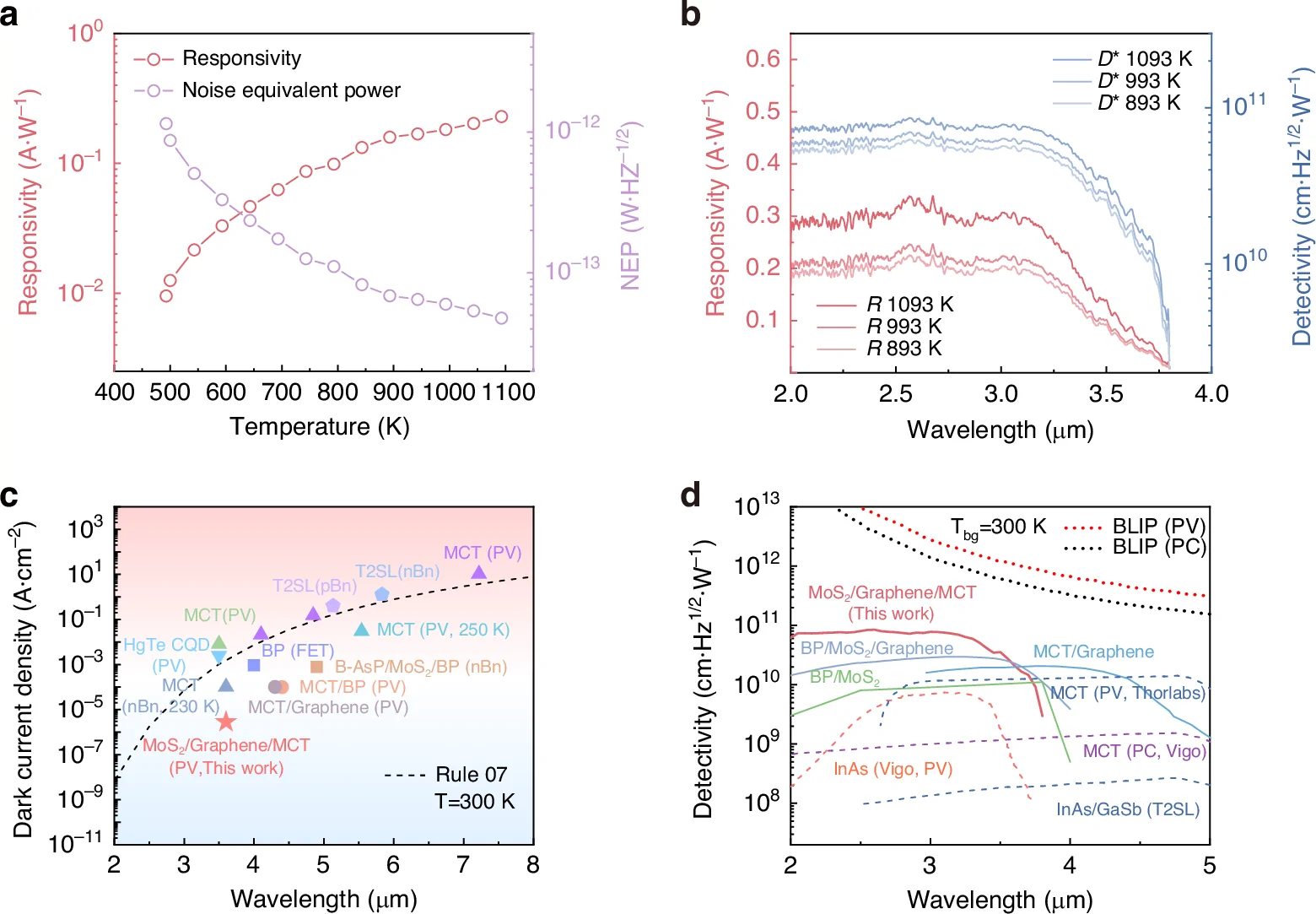
Blackbody characterization of MoS_2_/graphene/MCT vdW photodetectors at room temperature. **a** Responsivity and noise equivalent power of the MoS_2_/graphene/MCT vdW photodetectors under different blackbody temperatures. **b** Wavelength-dependent responsivity and specific detectivity at different blackbody temperatures measured at a background temperature of 300 K. **c** Dark current density comparison with the reported MWIR infrared photodetectors, including traditional detectors, 2D materials and 2D/MCT vdW photodetectors. **d** Spectral specific detectivity of the MoS_2_/graphene/MCT vdW heterostructure photodetector, other 2D materials-based MWIR photodetectors, and commercially available infrared photodetectors. PV photovoltaic, PC photoconductive, CQD colloidal quantum dot, T2SL type-Ⅱ superlattice

To assess the application potential of the photodetector, a blackbody source was used as a radiation source to simulate infrared detection conditions and characterize performance at room temperature. Schematic diagram of blackbody response measurement setup and output curve of MoS_2_/graphene/MCT vdW device under dark and 1093 K blackbody radiation are shown in Supplementary Fig. S22a, b. The substantial short-circuits current and open-circuit voltage generation reveal the typical photovoltaic mode for the blackbody radiation photoresponse. The temporal photoresponse of the device under different blackbody temperatures are shown in Supplementary Fig. S22c. As the temperature of the blackbody source decreases, the response amplitude of the photoresponse also diminishes. This phenomenon can be attributed to the drop of blackbody temperature induced radiation intensity reduction and the redshift of the peak wavelength (Supplementary Fig. S22d), which will result in a decreased responsivity and an increased noise equivalent power (NEP). Figure 5a shows the ${R}_{b}$ and NEP (${NEP}={i}_{n}/R$) of the photodetector at 300 K under different blackbody radiation temperatures, where ${i}_{n}$ is the root mean square current noise in a 1 Hz bandwidth. In the case of the incident light offered by blackbody radiation, the blackbody responsivity ${R}_{b}$ is given by ${R}_{b}={I}_{{ph}}/{P}_{b}$, where ${P}_{b}$ is the effective incident blackbody radiation power. The blackbody radiation power incident on the device was calculated using the approximate formula ${P}_{b}=\frac{\sigma \varepsilon \eta \left({T}_{b}^{4}-{T}_{a}^{4}\right){A}_{b}{A}_{D}}{\pi {L}^{2}}$, where σ is the Stefan-Boltzmann constant, ε is modulation factor, η is the average emissivity of the blackbody radiation source, ${T}_{b}$ is the temperature of blackbody source, ${T}_{a}$ is the ambient temperature, ${A}_{b}$ is the area of blackbody radiation aperture, and L is the distance between the radiation aperture and device. The MoS_2_/graphene/MCT vdW photodetector achieves a peak ${R}_{b}$ of 0.23 A W^−1^ and an ultra-low NEP of 4.8 × 10^−14^ W Hz^–1/2^ at 300 K under 1093 K blackbody radiation which is better than that of the recently reported room-temperature MWIR photovoltaic photodetectors based on 2D materials. Furthermore, the MoS_2_/graphene/MCT vdW structure can achieve blackbody detection across 493–1093 K, indicating its potential in weak signal recognition, while graphene/MCT heterojunction exhibit a poor measurement range limited by high dark current levels (Supplementary Fig. S22e, f). The Supplementary Fig. S23a presents the power-dependent ${R}_{b}$ and blackbody detectivity ${D}_{b}^{* }$ of the MoS_2_/graphene/MCT vdW photodetector under 1093 K blackbody radiation. The blackbody detectivity is estimated as ${D}_{b}^{* }={\left({A}_{D}{{\rm{B}}}\right)}^{1/2}{R}_{b}/{i}_{n}$. Under the identical blackbody temperature, the ${R}_{b}$ fluctuates from 0.21 A W^−1^ to 0.25 A W^−1^ as the radiation intensity varies from 18.1 mW cm^−2^ to 2.01 mW cm^−2^, and a high ${D}_{b}^{* }$ of the device is extracted as 7.0 × 10^10 ^cm Hz^1/2^ W^−1^ under diverse incident radiation power. Supplementary Fig. S23b, c compares the ${R}_{b}$ and ${D}_{b}^{* }$ of MoS_2_/MCT vdW heterojunction, graphene/MCT vdW heterojunction, and MoS_2_/graphene/MCT vdW photodetector under 1093 K blackbody radiation. The nearly an order-of-magnitude enhancement in ${D}_{b}^{* }$ stems from the synergy of graphene and MoS_2_ within the 2D/MCT vdW heterostructure, which improves photogenerated carrier collection efficiency and suppresses noise current. Blackbody peak photoresponsivity can be calculated according to formula ${R}_{P}={R}_{b}/g$, where *g* factors can be extracted from relative response spectrum *R*(λ) (Supplementary Fig. S24a). Figure 5b depicts the spectral blackbody peak responsivity and detectivity under diverse blackbody temperature radiations. The blackbody peak photoresponsivity at 1093 K, 993 K and 893 K were 0.33 A W^−1^, 0.24 A W^−1^ and 0.22 A W^−1^, respectively, with an average specific detectivity in MWIR spectrum up to 8.0 × 10^10^ cm Hz^1/2^ W^−1^ under 1093 K blackbody radiation, indicating an excellent detection performance among the existing MWIR photodetectors. Supplementary Fig. S24b displays the blackbody peak responsivity and specific detectivity across a range of blackbody temperatures from 493 K to 1093 K and a peak specific detectivity is evaluated as high as 8.9 × 10^10^ cm Hz^1/2^ W^−1^ under 1093 K blackbody radiation.

To further demonstrate the superiority of the MoS_2_/graphene/MCT vdW photodetector, the performance comparison of our device and other reported MWIR photodetector is show in Fig. 5c, d and Supplementary Table S1. Figure 5c summarizes the spectrum-dependent dark current density of diverse traditional MWIR photodetectors under working mode at room temperature, such as MCT photodiode^9,10^, type-Ⅱ InAs/InAsSb superlattice (T2SL)^35,36^, nBn structure^11^, colloidal quantum dots (CQD) photodetector^37^, and advanced 2D materials-based photodetector^38,39^. The dashed line defined by empirical equation Rule 07 is included for comparison and indicates the dark current level of advanced MCT photodiodes. Benefitting from the rational 2D/MCT vdW structure design, our 2D/MCT vdW device achieves the lower dark current density when operating at room temperature, demonstrating its capability for weak-signal recognition without cryogenic cooling. The room-temperature blackbody detectivity of the MoS_2_/graphene/MCT vdW photodetector as a function of the wavelength is compared with commercial photodetectors and 2D materials-based photodetectors in Fig. 5d and Supplementary Table S1^18,40,41^. The blackbody detectivity and other figures of merit of the proposed 2D/MCT vdW photodetector are comparable to those of the most advanced commercial photodetectors, indicating that MoS_2_/graphene/MCT vdW photodetectors could have promising prospects in uncooled state-of-the-art MWIR photodetection.

## Discussion

In conclusion, we have designed and fabricated an uncooled high-performance MWIR photodetector based on a MoS_2_/graphene/MCT tri-layered vdW structure. The incorporation of graphene into the 2D/MCT vdW heterostructure synergistically blocked dark current and suppressed interfacial recombination, leading to extended carrier lifetime and efficient interlayer charge transfer. This results in a 2D/MCT vdW photodetector with impressive performance metrics, including a peak responsivity *R*_p_ of 0.325 A W^−1^ and an ultra-low NEP of 0.048 pW Hz^−1/2^ at room temperature. Furthermore, the device achieves an impressive specific detectivity of 8.0 × 10^10^ cm Hz^1/2^ W^–1^ under 1093 K blackbody radiation, representing nearly an order of magnitude improvement in sensitivity compared to counterpart devices. The performance of our device is comparable to that of commercially available infrared photodetectors and advanced 2D-material-based infrared photodetectors indicating the superior device architecture of our 2D/MCT vdW heterostructure. Our work highlights that van der Waals/MCT heterostructures can be a critical solution to overcome dark current and interfacial recombination in MCT-based devices, providing a design paradigm for next-generation uncooled high-performance infrared photodetectors.

## Materials and methods

### Device fabrication

MCT thin film with a thickness of 8 μm was epitaxially grown on a ZnCdTe substrate by liquid phase epitaxy, and the MCT wafer was etched in a bromoethanol solution for 10 s to remove the surface defects caused by growth or oxidation. The polymethacrylate (PMMA) was spin-coated on the MCT surface for electrical isolation, and a 30 × 30 μm^2^ window was exposed by electron beam lithography (EBL) and developer. The multilayer MoS_2_ and graphene were mechanically exfoliated from the bulk material and transferred to the exposed MCT window to fabricate heterostructure via dry transfer technique. After that, a thin layer of water-soluble polyvinyl acetate (PVA) with pre-patterned Au electrode was put on a layer of transparent elastic stamp polydimethylsiloxane (PDMS) and transferred to the sample surface. The PVA film was dissolved by soaking the sample in deionized water during the lift-off process.

### Pump−probe spectra measurements

The laser pulses were produced by Ti: sapphire oscillators (800 nm, 80 MHz, 140 fs). One beam was set to a fixed wavelength of 800 nm, and the other was used to drive the optical parametric oscillator, producing pulses with the wavelength from 1000 to 1500 nm. Both the pump and probe laser were focused onto the sample by a 50× objective lens, and the pump fluence was 10 times higher than the probe fluence. The delay between the pump and probe pulses was controlled by a delay line (Newport, DL325) with a step length of 20 μm, corresponding to a time delay step of 133 fs. The chopper (Stanford Research Systems, SR540) frequency was fixed to 1 kHz as the reference frequency of the lock-in amplifier (Stanford Research Systems, SR830). Changes in reflectivity of the probe laser induced by the pump laser was detected using a balanced amplified photodetector. (Thorlabs, PDB 210A).

### Characterizations and measurements

All electrical and optoelectrical measurements were performed at room temperature. The Raman spectra and photoluminescence measurements were carried out on LabRAM HR800 micro-Raman system (Horiba Jobin Yvon) with a 532 nm laser. The photoresponse characteristics from visible to SWIR were measured using an fs-laser source (Chameleon Compact OPO, center wavelength: 900 nm, repetition rate: 80 MHz), focused on the active area by a 50× objective lens. The mid-wavelength infrared response is measured by a tunable OPO laser (Cati-T-3-4) and electrical signals generated by the device were collected and analyzed using a source meter (FS-Pro, PRIMARIUS). The blackbody response measurement was performed using an HFY-200BII calibrated commercial blackbody source and the diameter of the aperture was 10 mm. The thickness of 2D materials and contact potential difference were carried out on Scanning Kelvin Probe Microscopy (Oxford Instrument MFP-3D). Photocurrent mapping was performed by WITEC (alpha 300R). The photoresponse time of the device was tested using 1550 nm laser coupled with a high-speed electro-optic modulator (MXAN-LN-40), a digital oscilloscope (Tektronix MDO3032), switchable current amplifier (DHPCA-100), vector network analyzer (Seyear 3672C) and wide bandwidth amplifier (SHWWA-00014000-14P20).

## Supplementary information

## Data Availability

The data that support the findings of this study are available from the corresponding author upon reasonable request.
