# Supplementary material for: Van der Waals/MCT heterostructure enabled high-performance uncooled mid-infrared photodetectors via synergistic suppression of dark current and interfacial recombination
Source: Light Sci Appl. 2026 Sep 2;15:367. doi: 10.1038/s41377-026-02418-y (PMC13538648; doi:10.1038/s41377-026-02418-y)
Supplement: Supplementary file 1 [file 41377_2026_2418_MOESM1_ESM.pdf]

Supplementary Information for

Van der Waals/MCT Heterostructure Enabled High-Performance Uncooled Mid-Infrared Photodetectors via Synergistic Suppression of Dark Current and Interfacial Recombination

Xinlei Zhang[#1], Tao Zhou[#1], Yueying Cui[1], Ruizhi Li[1], Hao Wu[1], Yuwei Zhang[1], Rui Lin[1], Fang Yang[2], Kaiyang Liu[1], Jianfeng Wu[3], Dongyang Wan*[1], Jialin Zhang*[1], Junpeng Lu[1,4,5], Zhenhua Ni*[1,4]

[1] Key Laboratory of Quantum Materials and Devices of Ministry of Education, School of Physics, Southeast University, Nanjing 211189, China

[2] Key Laboratory of State Manipulation and Advanced Materials in Provincial Universities, School of Physics and Technology, Nanjing Normal University, Nanjing 210023, China

[3] School of Integrated Circuits, Southeast University, Nanjing 210096, China

[4] School of Electronic Science and Engineering, Southeast University, Nanjing 210096, China

[5] Advanced Ocean Institute, Southeast University, Nanjing 210096, China

Authors to whom correspondence should be addressed: wandy@seu.edu.cn, phyzjl@seu.edu.cn, zhni@seu.edu.cn

[#] Xinlei Zhang and Tao Zhou contributed equally to this work

Supplementary Note S1. Band structure and trap states calculations of HgCdTe

Calculation of component and band structure of HgCdTe

Component x of $Hg_{1-x}Cd_xTe$ is estimated via the formula proposed by Chu as follows:[1]

$$E_g = -0.295 + 1.87x - 0.28x^2 + (6 - 14x + 3x^2)10^{-4}T + 0.35x^4(eV) \quad (1)$$

where E_g is the energy bandgap of $Hg_{1-x}Cd_xTe$, x is the component of cadmium, and T is the temperature. Since the cutoff wavelength (λ_c) is estimated as ~3.6 μm from Fig. S2a, the $E_g = 0.35\ eV$ is extracted via equation $E_g = \frac{hc}{\lambda_c}$ at room temperature (300 K), Here, λ_c is defined as the wavelength where absorption decreases to 50% of the maximum value. Thus, the component x is calculated as around 0.33 according to equation (1).

The conduction band minimum (CBM) of $Hg_{1-x}Cd_xTe$ can be extracted from the CBM upward offset (ΔE_v) equation $\Delta E_c = 1.21(1 - x)$ from CdTe semiconductors.[2] Since the CBM of CdTe at room temperature is reported as 3.8 eV,[3] the CBM of HgCdTe in this work can be calculated as 4.610 eV. Hence, the valance band maximum (VBM) could be estimated via formula $E_v = E_c + E_g$ as 4.960 eV.

The Fermi level according to the following formulas:

$$N_p = N_v \exp\left(-\frac{E_F - E_v}{k_B T}\right) \quad (2)$$

$$N_v = 2\left(-\frac{m_h^* k_B T}{2\pi h^2}\right)^{3/2} \quad (3)$$

where N_P is the free hole volume concentration, N_v is the valance band equivalent density of states, $E_F - E_v$ is the distance from Fermi level to CBM, respectively, k_B is the Boltzmann constant, T is the temperature, m_h^* is the hole effective mass. For the commonly used component of $Hg_{1-x}Cd_xTe$ (0.2-0.5), the effective mass of MCT is $0.55m_0$. The doping concentration of MCT (N_P) is $\sim 10^{18}$ cm^{-3} at room temperature from Fig. S2b. We substituted N_P into formula (3) to figure out ($E_V - E_F$) to be 10 meV for MCT.

Calculation of shallow and deep trap states within $Hg_{1-x}Cd_xTe$

As a ternary alloy compound, HgCdTe (MCT) has a zinc blende lattice structure. The neutral mercury vacancy (V_{Hg}) has six electrons from its Te neighbors, so it tends to accept one or two more electrons, producing the singly or doubly negatively charged vacancy levels with higher energy than for the neutral state. The neutral V_{Hg} state as a shallow acceptor whose location has been well defined.[4] The ionization energy for the material composition (x value) from 0.18 to 0.3 can be calculated from the relation:[5]

$$E_1(x) = 91.1x - 6.93\ (meV) \tag{4}$$

where x is the component of cadmium. This means that the acceptor level is located near the top of the valence band maximum. For $x \sim 0.3$, the second level can be treated as a deep defect state located in the mid of the energy gap. The ionization energy of the second defect state which probably originating from the singly negatively charged V_{Hg}:[5]

$$E_2(x) = 144x + 56\ (meV) \tag{5}$$

The Shockley-Read-Hall (SRH) recombination, occurring through deep defect levels within the bandgap, is responsible for minority carrier lifetimes in lightly-doped MCT. At high doping concentrations, these deep centers can facilitate SRH current and trap-assisted tunneling of carriers across the junction, which will elevate the dark current and hinder the development of high-operating-temperature (HOT) MCT devices.[6]

According to the shallow and deep trap states calculation formula, the shallow and deep trap states for $Hg_{1-x}Cd_xTe$ (x~0.33) are located at 24 meV and 104 meV above the valence band, respectively.

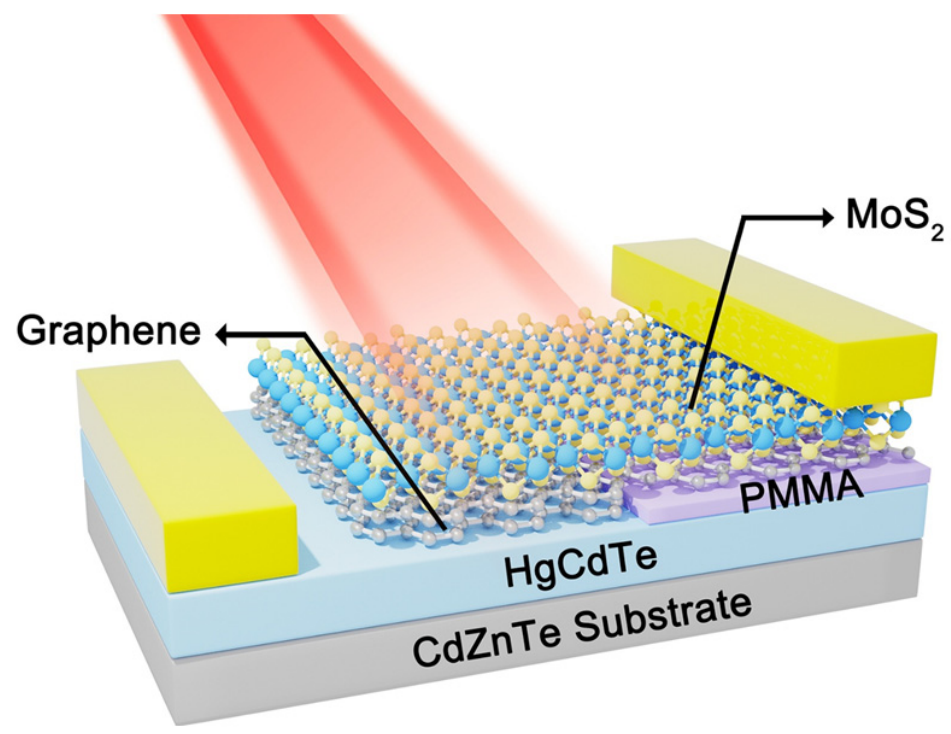

Fig. S1. Schematic diagram of the MoS_2/graphene/MCT van der Waals (vdW) heterostructure photodetector.

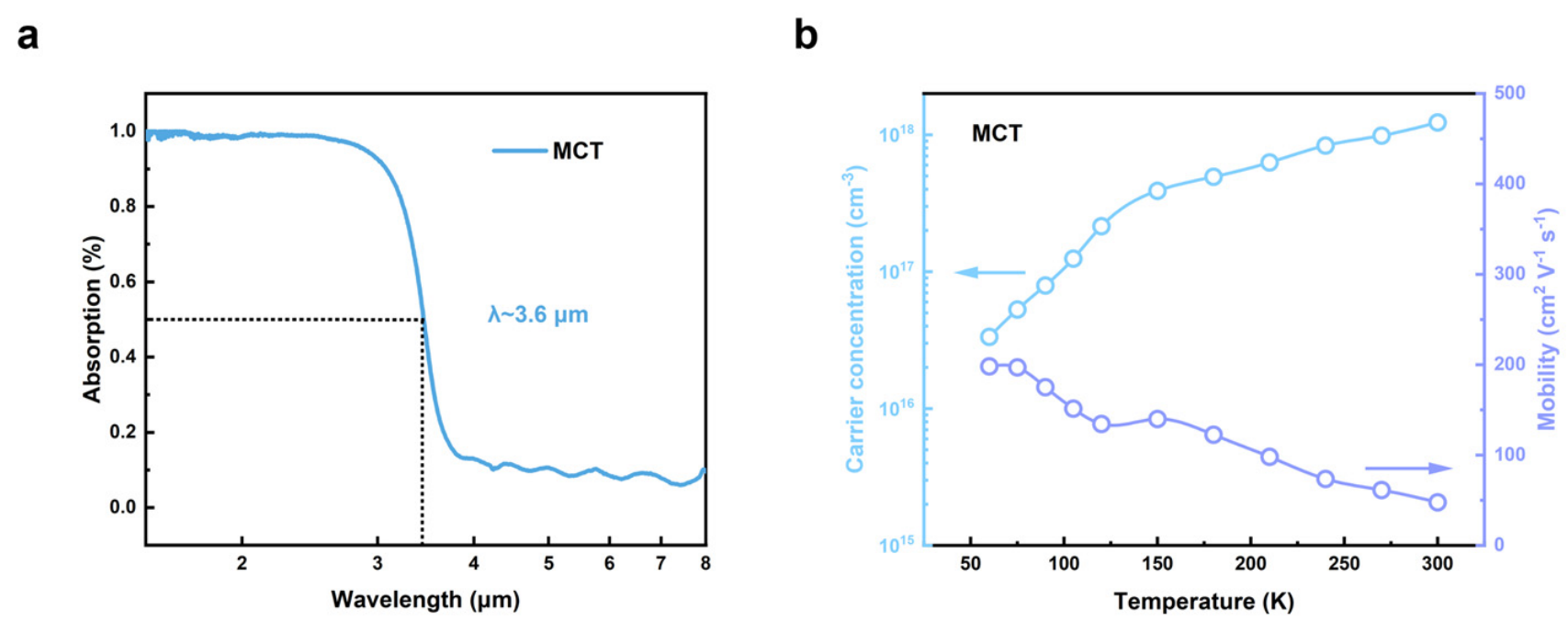

Fig. S2. Optical and electrical characterization of HgCdTe film. **a** Infrared absorption spectra of HgCdTe film. The cutoff wavelengths λ_c of HgCdTe is ~3.6 μm. **b** Hall measurement results of HgCdTe film. With the decrease of temperature, the carrier mobility of HgCdTe increases, the carrier concentration decreases and conduction type remains p-type.

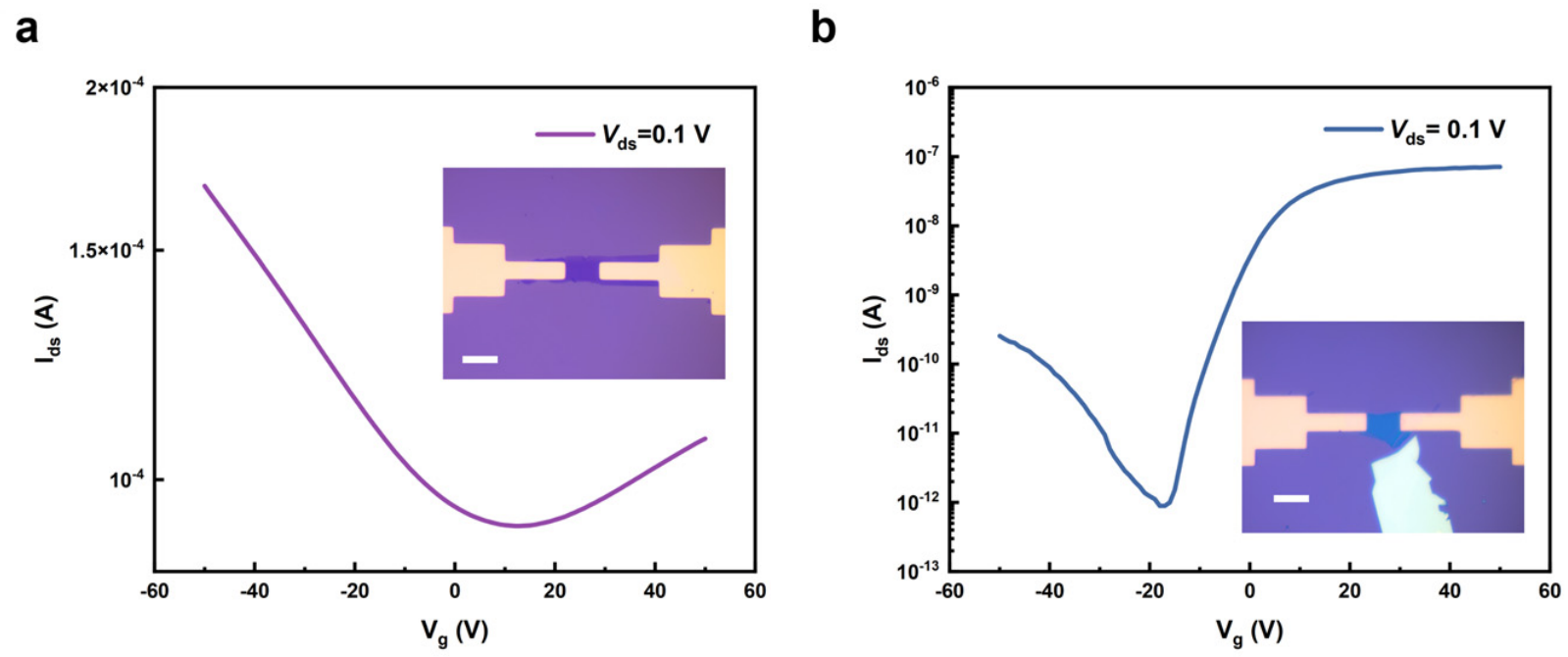

Fig. S3. Electrical characterization of graphene and MoS_2. **a** Transfer characteristic of the graphene field-effect transistor (FET) device. Inset is the optical microscope image; scale bar is 20 μm. **b** Transfer characteristic of the MoS_2 FET device. Inset is the optical microscope image; scale bar is 20 μm.

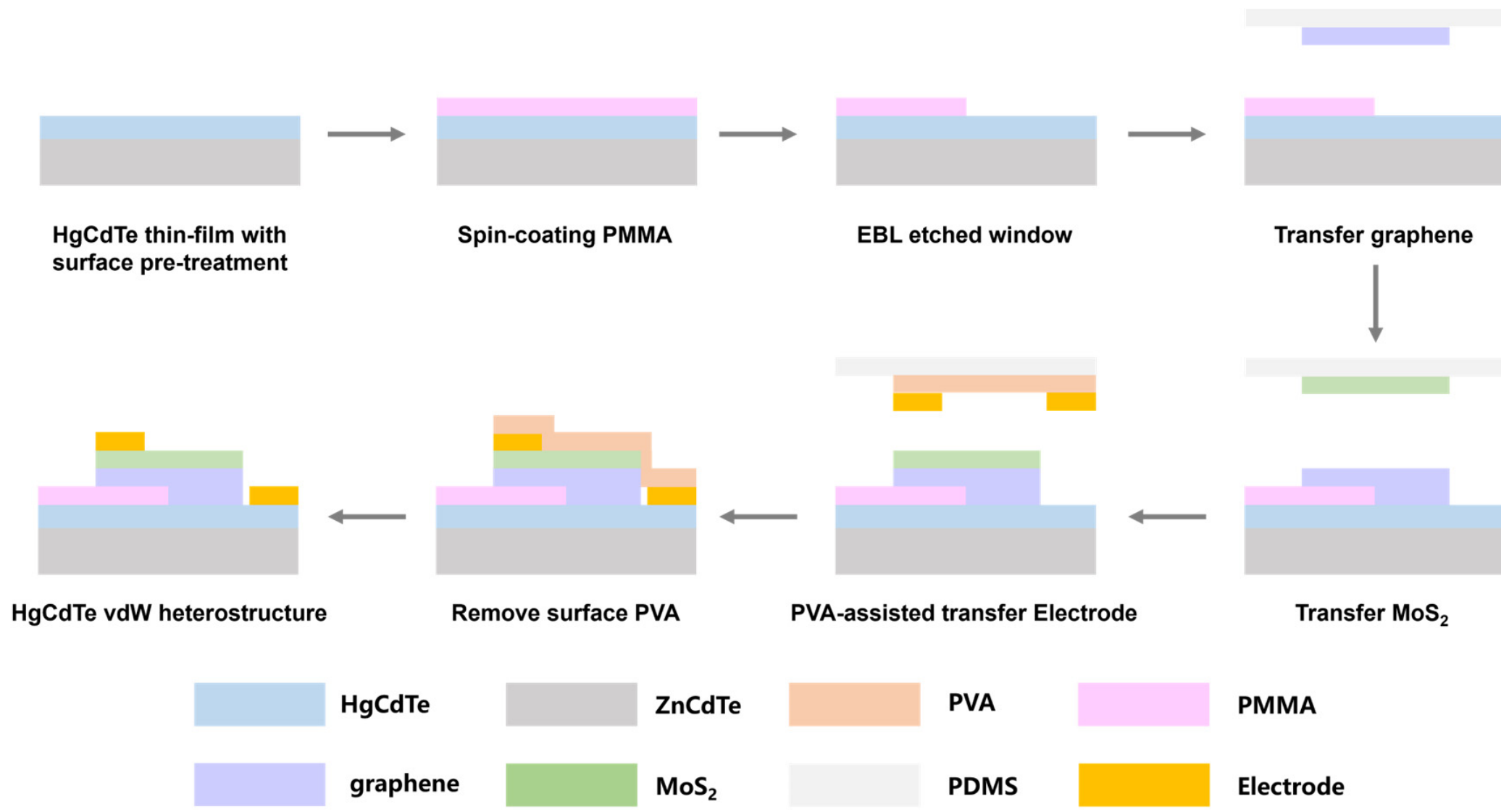

Fig. S4. Fabrication steps of MoS_2/graphene/MCT vdW heterostructure device.

Initially, all experimental samples were fabricated on the same batch of liquid phase epitaxy (LPE) grown HgCdTe (MCT) epitaxial wafers (grown on ZnCdTe substrates) to guarantee the consistency of carrier concentration and material composition. To eliminate surface defects induced by the growth process or natural oxidation, the MCT wafers underwent a chemical etching treatment in a bromoethanol solution for 10 s. Subsequently, a layer of polymethacrylate (PMMA) was spin-coated onto the cleaned MCT surface to serve as an electrical isolation layer. Using electron beam lithography (EBL) process, an active window of $30 \times 30\ \mu m^2$ was precisely exposed, thereby strictly defining the effective working area for the various heterostructure devices. Following this, high-quality few layer graphene and MoS_2 flakes were obtained from bulk crystals via mechanical exfoliation and were sequentially stacked onto the exposed MCT window using a precise dry transfer technique to construct the van der Waals (vdW) heterostructures. Then, the pre-patterned Au electrodes were picked-up onto a water-soluble polyvinyl acetate (PVA)/polydimethylsiloxane (PDMS) transparent elastic stamp, which was then aligned and transferred onto the sample surface. After transfer, the samples were immersed in deionized water to dissolve the PVA layer and complete the lift-off process, yielding the final MoS_2/graphene/MCT vertical heterostructure devices.

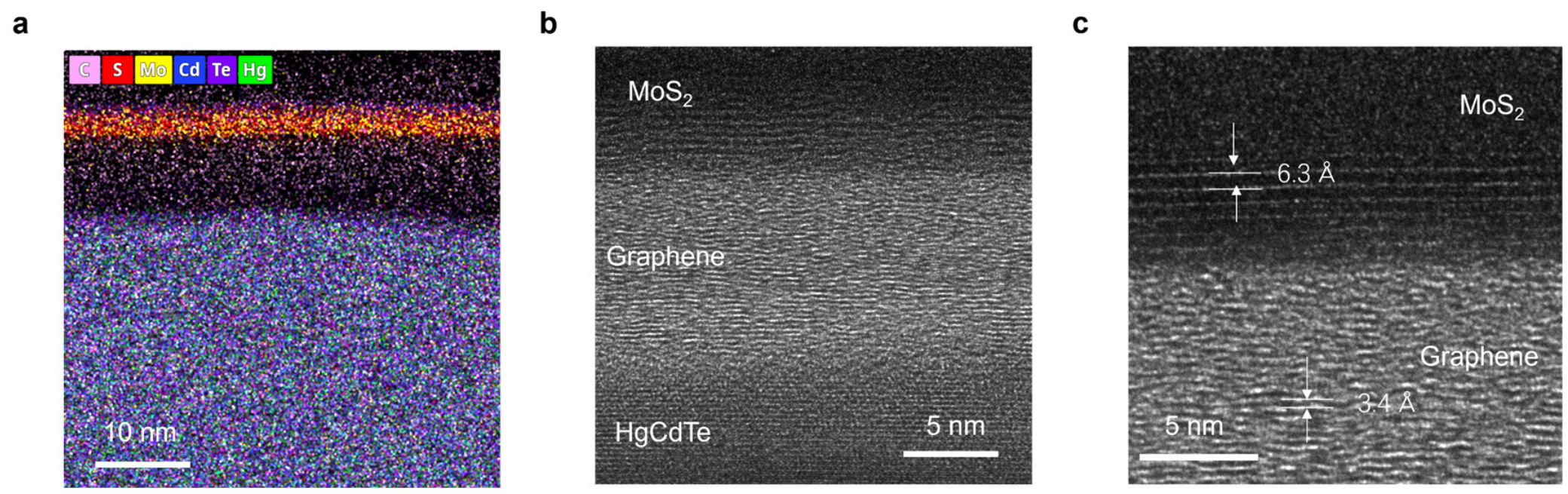

Fig. S5. Cross-sectional TEM image of the vdW/MCT heterostructure. **a** The energy-dispersive X-ray spectroscopy (EDX) mapping of the cross section of a MoS_2/graphene/MCT heterostructure. **b** High-resolution TEM image of the MoS_2/graphene/MCT and **c** MoS_2/graphene cross section.

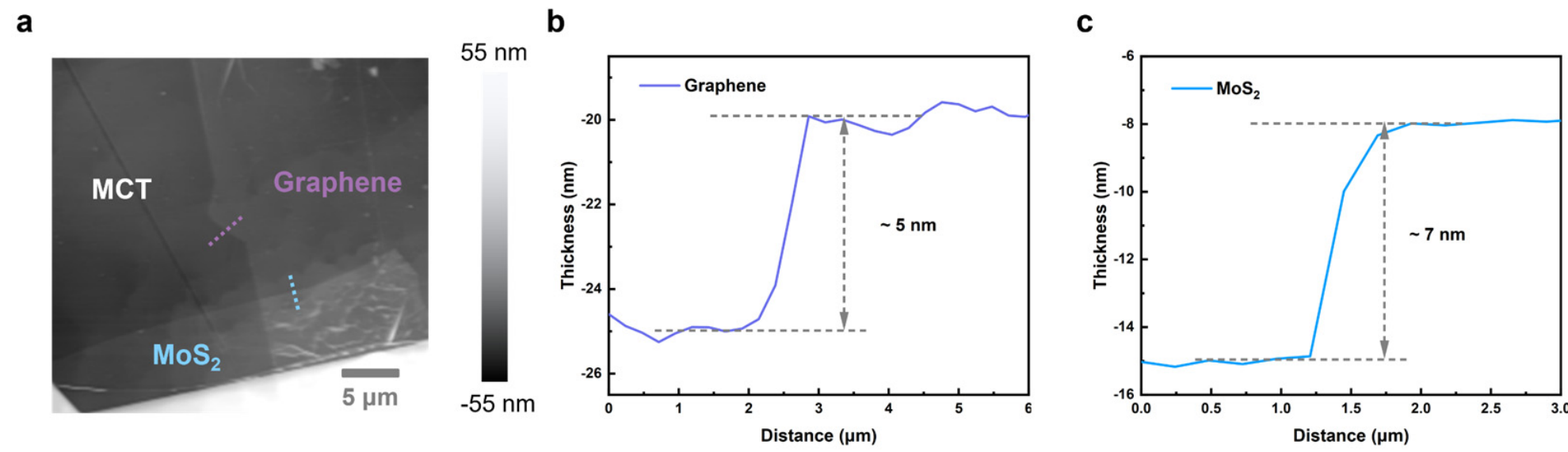

Fig. S6. Thickness characterization of the tri-layered MoS_2/graphene/MCT vdW heterostructure. **a** AFM mapping image of the heterostructure. The scale bar is 5 μm. The thicknesses of **b** graphene and **c** MoS2, extracted from Figure S6a, are 5 nm and 7 nm, respectively.

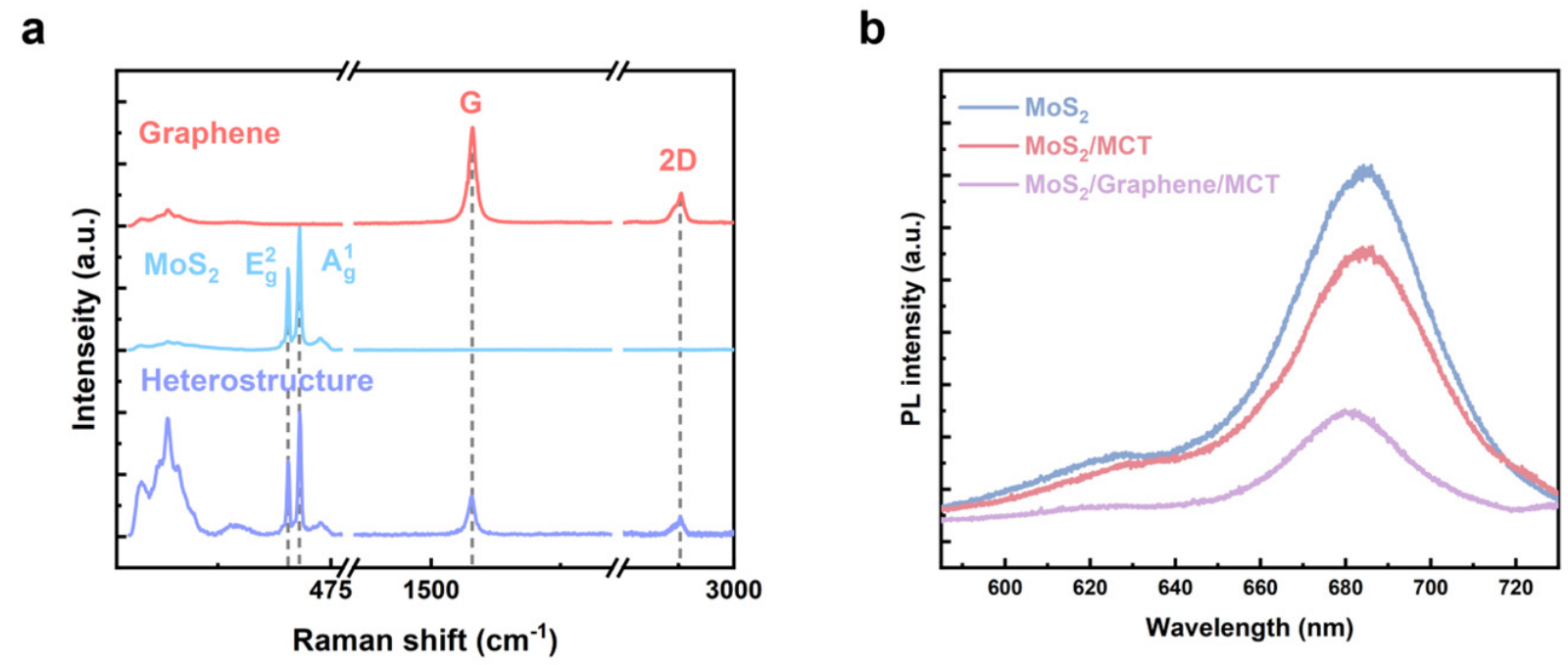

Fig. S7. Raman spectrum and Photoluminescence (PL) spectrum of tri-layer 2D/MCT vdW heterostructure. **a** Raman spectrum of the graphene, MoS_2, and overlap of the heterostructure. **b** PL spectra of the MoS_2/graphene/MCT vdW heterostructure, MoS_2/MCT heterostructure, and pristine MoS_2 under 532 nm excitation source.

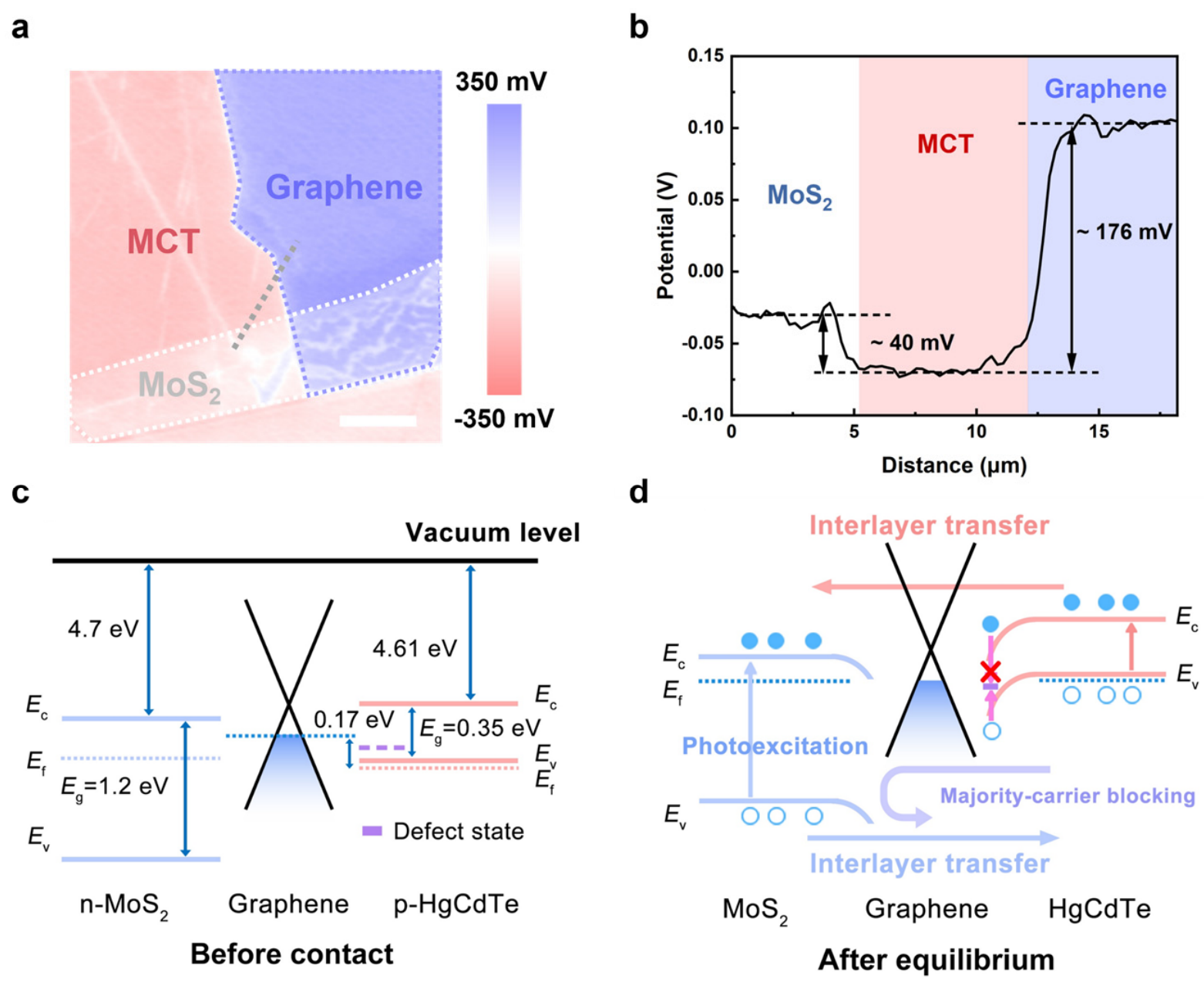

Fig. S8. Surface potential characterization and band alignment of tri-layer 2D/MCT vdW heterostructure. **a** Surface potential mapping image of the MoS_2/graphene/MCT heterostructure, the scale bar is 15 μm. **b** Surface potential difference between MoS_2, MCT and graphene along the gray dashed line from surface potential mapping. **c** Energy band profiles of MoS_2/graphene/MCT heterostructure. **d** Schematic diagram of charge transfer in the MoS_2/graphene/MCT heterostructure.

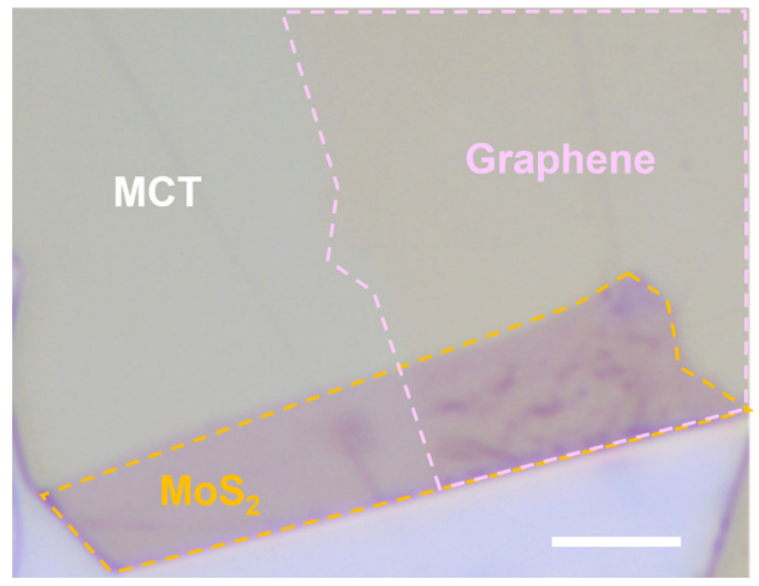

Fig. S9. Optical microscope image of MoS_2/graphene/MCT vdW heterostructure, the scale bar is 15 μm.

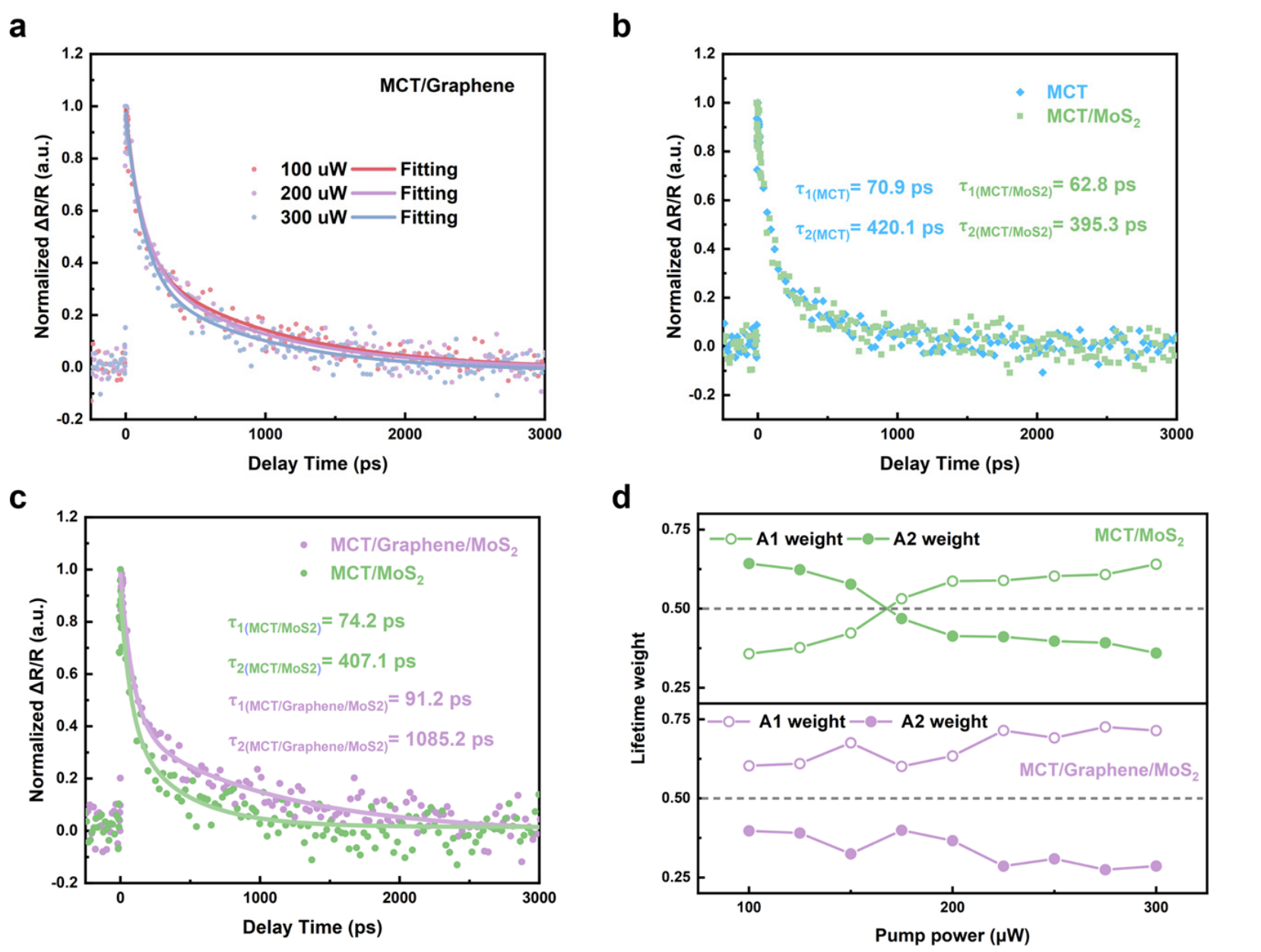

Fig. S10. Pump-probe characterization of graphene/MCT heterostructure, MoS_2/MCT heterostructure, and MoS_2/graphene/MCT vdW structure. **a** Power-dependent transient reflection spectra and fitting curve of MCT/graphene heterostructure. **b** Carrier dynamics comparison of the MoS_2/MCT heterostructure and pristine MCT. **c** Carrier dynamics comparison of the MoS_2/MCT heterostructure and MoS_2/graphene/MCT vdW structure. **d** Power-dependent lifetime weight comparison of MoS_2/MCT heterostructure and MoS_2/graphene/MCT vdW structure.

The similar decay profiles and power-dependent lifetime weight observed in MoS_2/MCT heterostructure and pristine MCT (Fig. S10b) indicated MoS_2 hardly affect the carrier recombination process within MCT. This can be ascribed to the small work difference between the MCT and MoS_2 and low carrier concertation of MoS_2. The passivation effect of graphene is also reflected in the MoS_2/MCT vdW heterostructure as observed extended lifetime and different power-dependent lifetime weight (Fig. S10c-d).

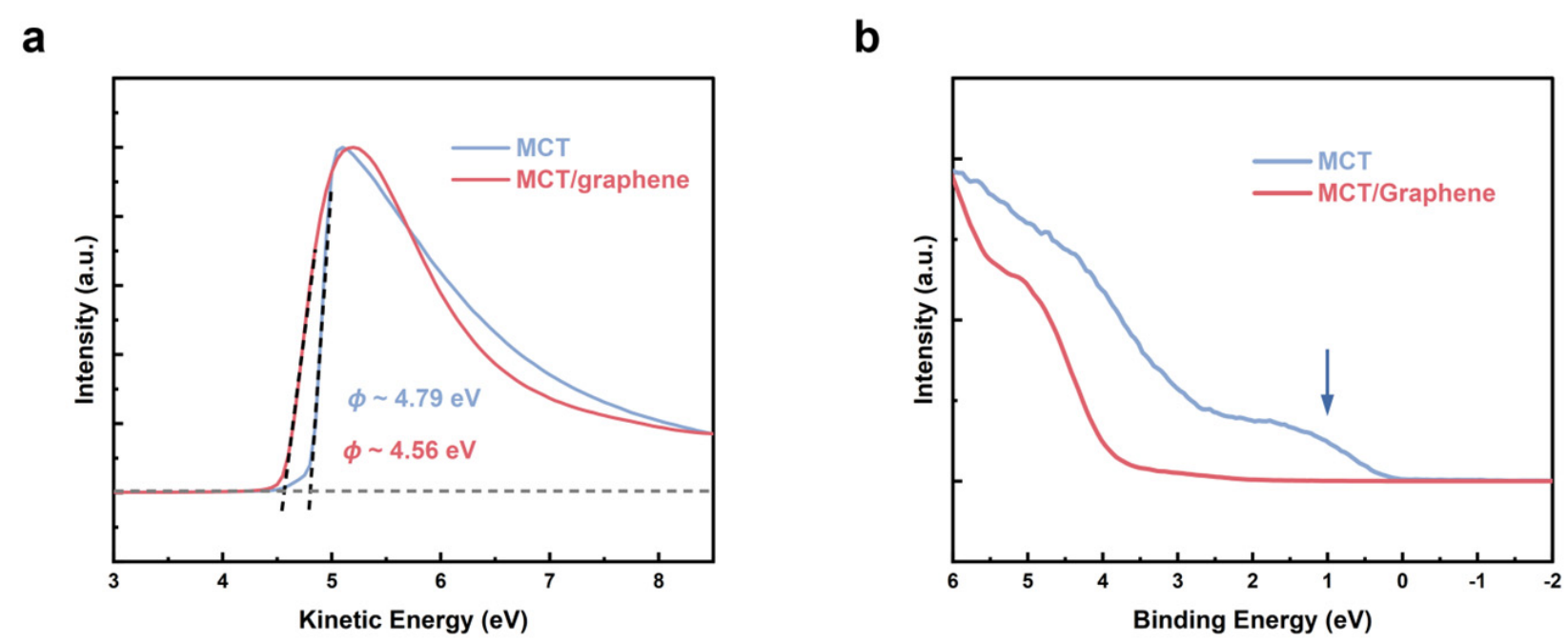

Fig. S11. Ultraviolet photoelectron spectroscopy (UPS) characterization and comparison of MCT and MCT/graphene heterostructure. **a** UPS spectra comparison of secondary electron cutoff region between the MCT and graphene/MCT heterostructure. **b** UPS spectra comparison of valance band region between the MCT and graphene/MCT heterostructure.

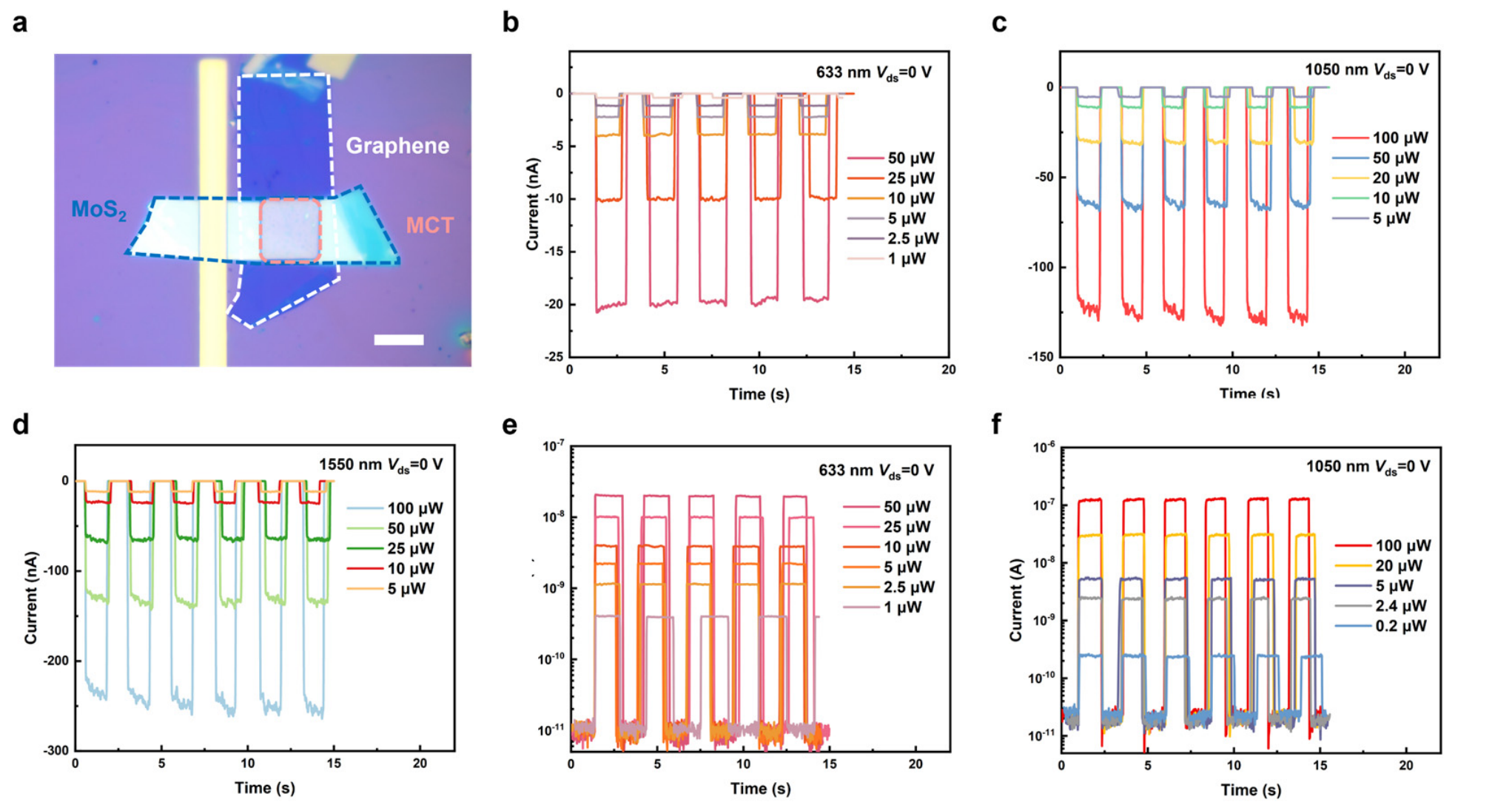

Fig. S12. Photoresponse of the MoS_2/graphene/MCT vdW photodetector under visible and near-infrared laser illumination. The scale bar is 30 μm. **a** Optical image of the MoS_2/graphene/MCT vdW photodetector. **b-d** Time-resolved photoresponse of the MoS_2/graphene/MCT vdW photodetector under various power laser illumination with the wavelengths of 633 nm, 1050 nm, 1550 nm in linear scale and **e-f** in logarithmic scale.

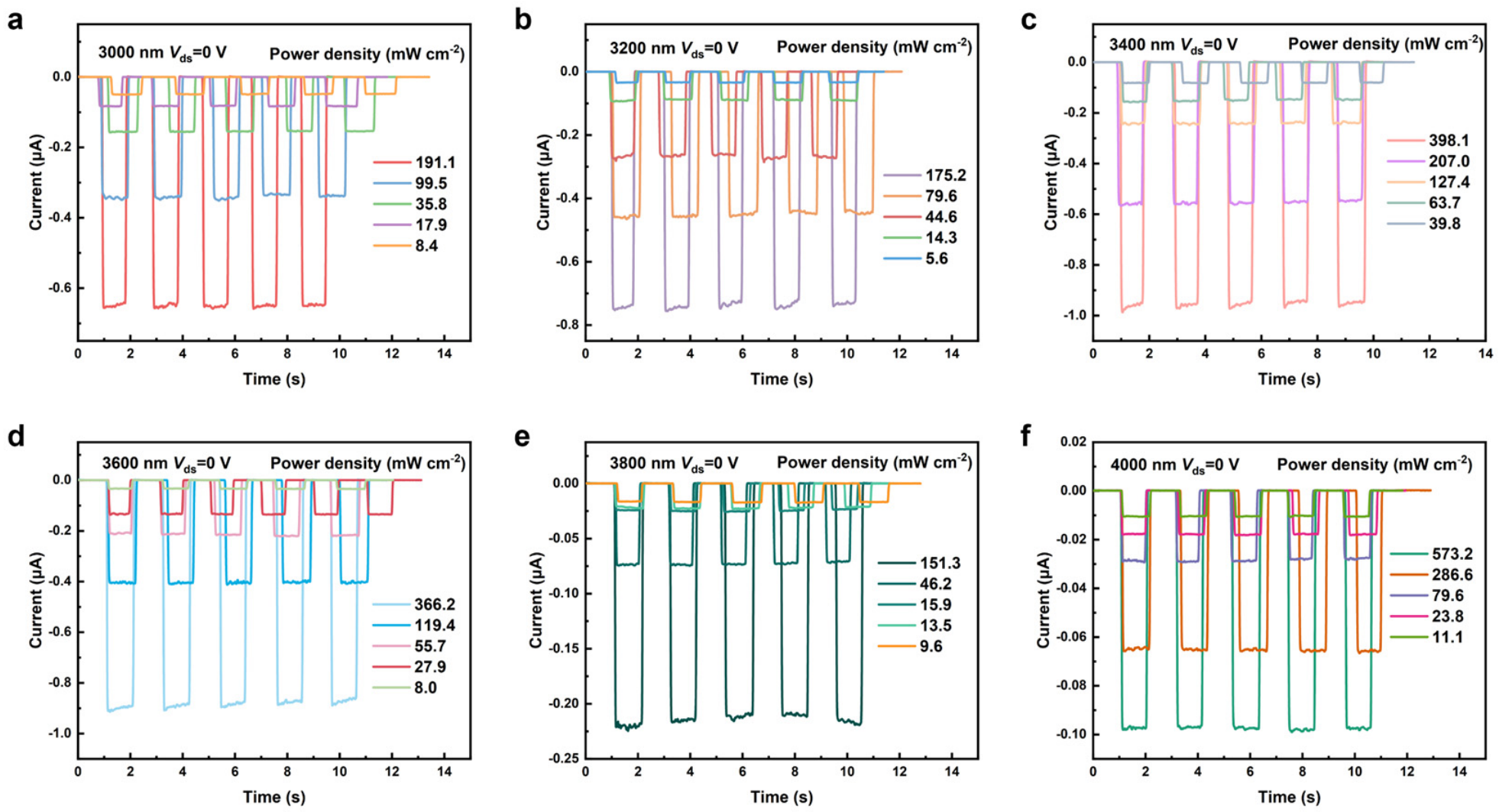

Fig. S13. Photoresponse of the MoS_2/graphene/MCT vdW photodetector under MWIR laser illumination. **a-f** Time-resolved photoresponse of the MoS_2/graphene/MCT vdW photodetector under various power laser illumination with the wavelengths of 3000 nm, 3200 nm, 3400 nm, 3600 nm, 3800 nm, and 4000 nm.

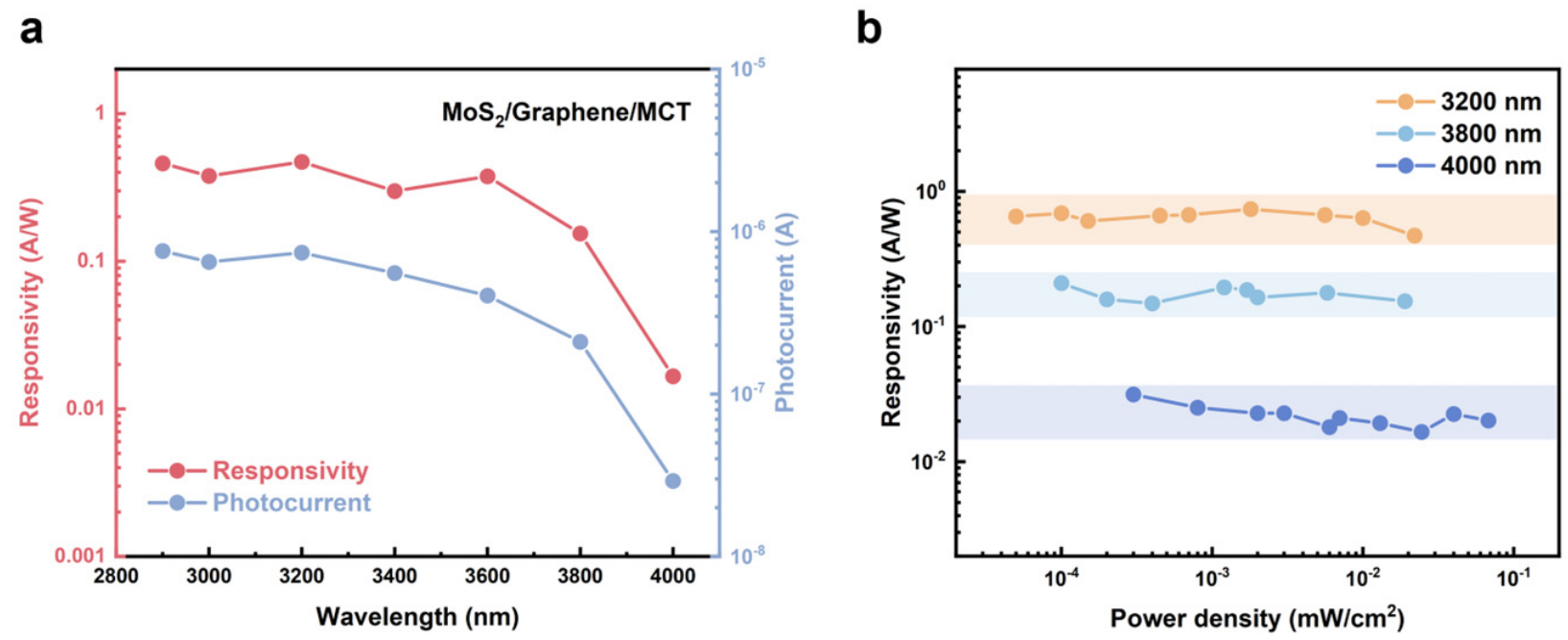

Fig. S14. Photoresponsivity of the MoS_2/graphene/MCT vdW photodetector under the MWIR laser irradiation at V_{ds}=0 V. **a** Wavelength-dependent responsivity and photocurrent of the MoS_2/graphene/MCT vdW photodetector under ~20 mW cm^{-2} MWIR illumination. **b** Power-dependent photoresponsivity of the MoS_2/graphene/MCT vdW photodetector under 3200 nm, 3800nm, and 4000 nm laser irradiation.

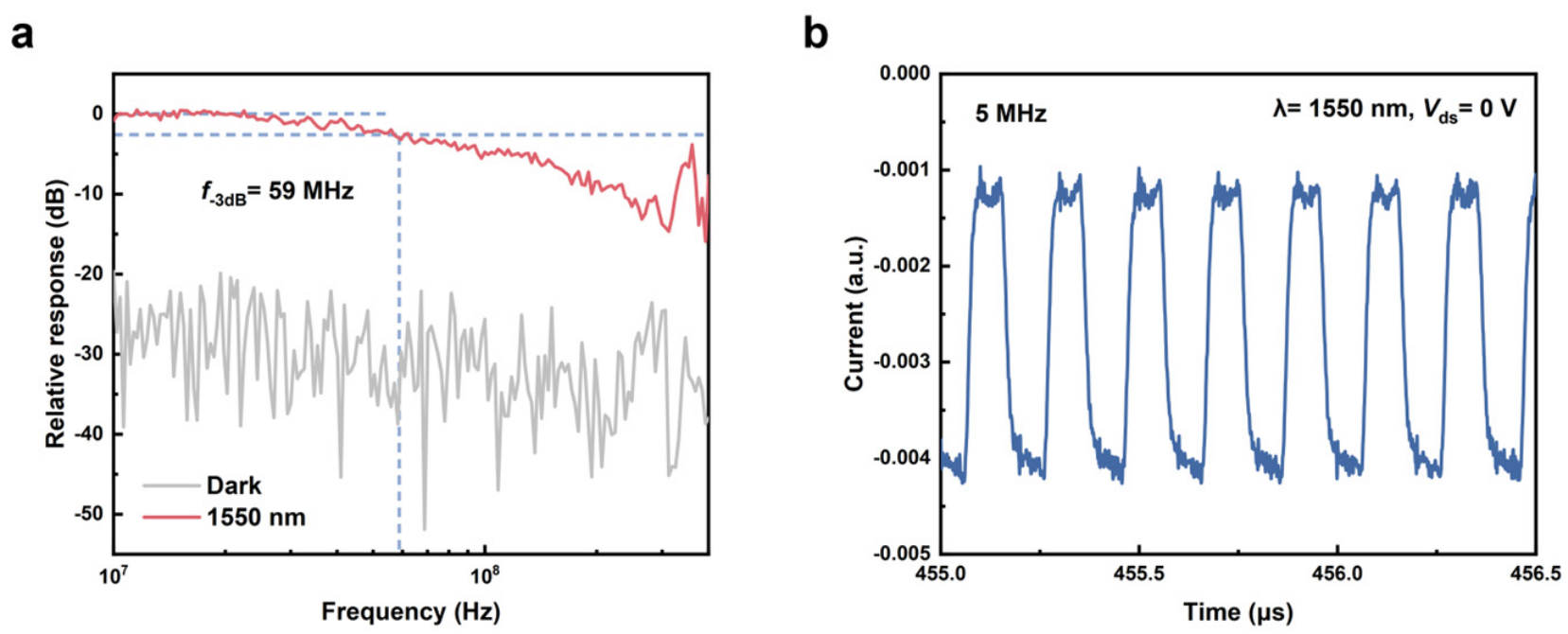

Fig. S15. Response time characterization via high-speed vector network analyzer test system. **a** Relative response versus switching frequency, showing the -3 dB cutoff frequency of 59 MHz. **b** Time resolved photoresponse of MoS_2/graphene/MCT device under 1550 nm illumination at 5 MHz frequency.

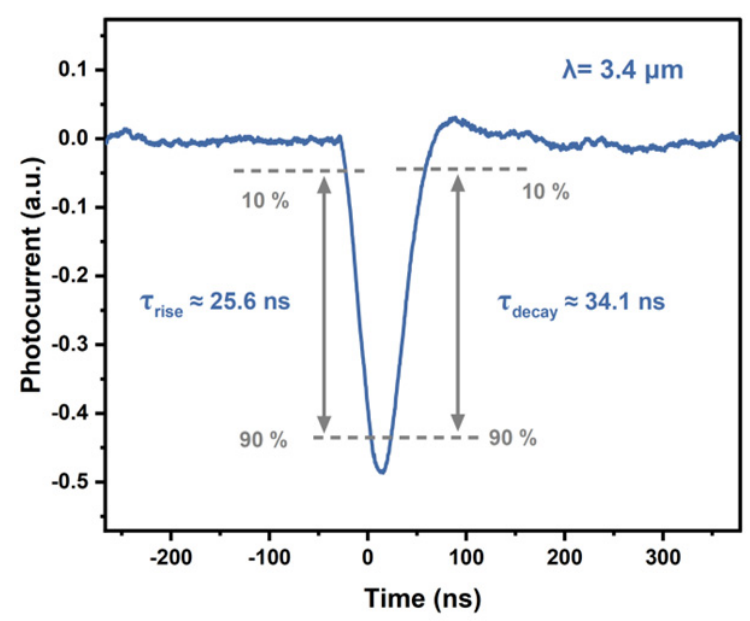

Fig. S16. Time-resolved photoresponse of MoS_2/graphene/MCT vdW photodetector under 3400 nm ultrafast pulsed lasers.

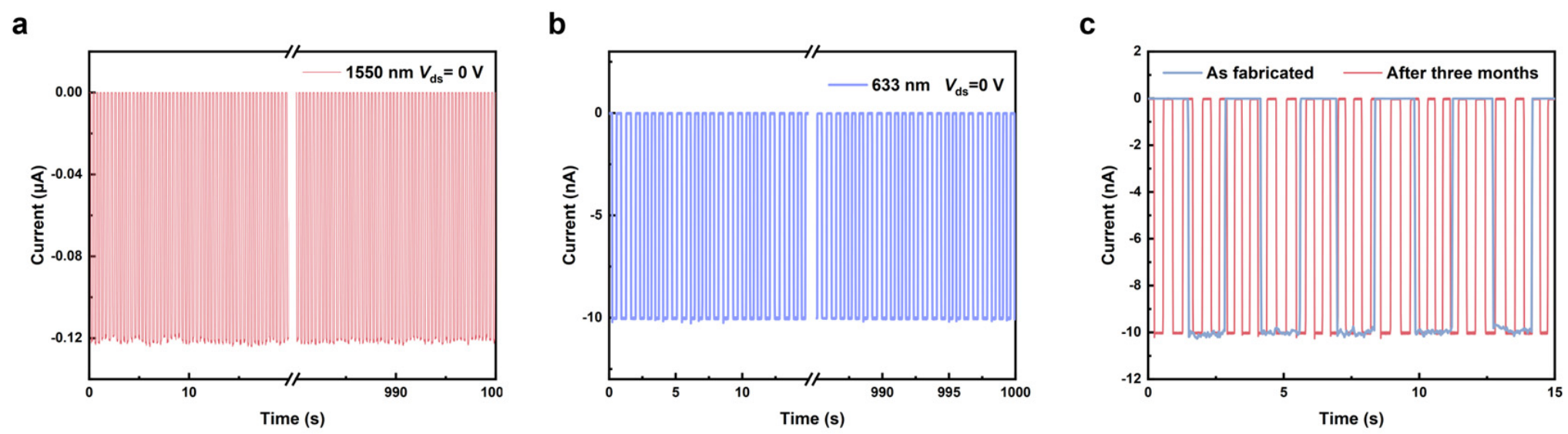

Fig. S17. Stability of the MoS_2/graphene/MCT vdW photodetector. **a**, Continuous operation of the vdW/MCT photodetector under 633 nm and **b**, 1550 nm at $V_{ds} = 0$ V. **c**, Time-resolved photoresponse for as fabricated device and the same device after three months storage at a wavelength of 633 nm at $V_{ds} = 0$ V.

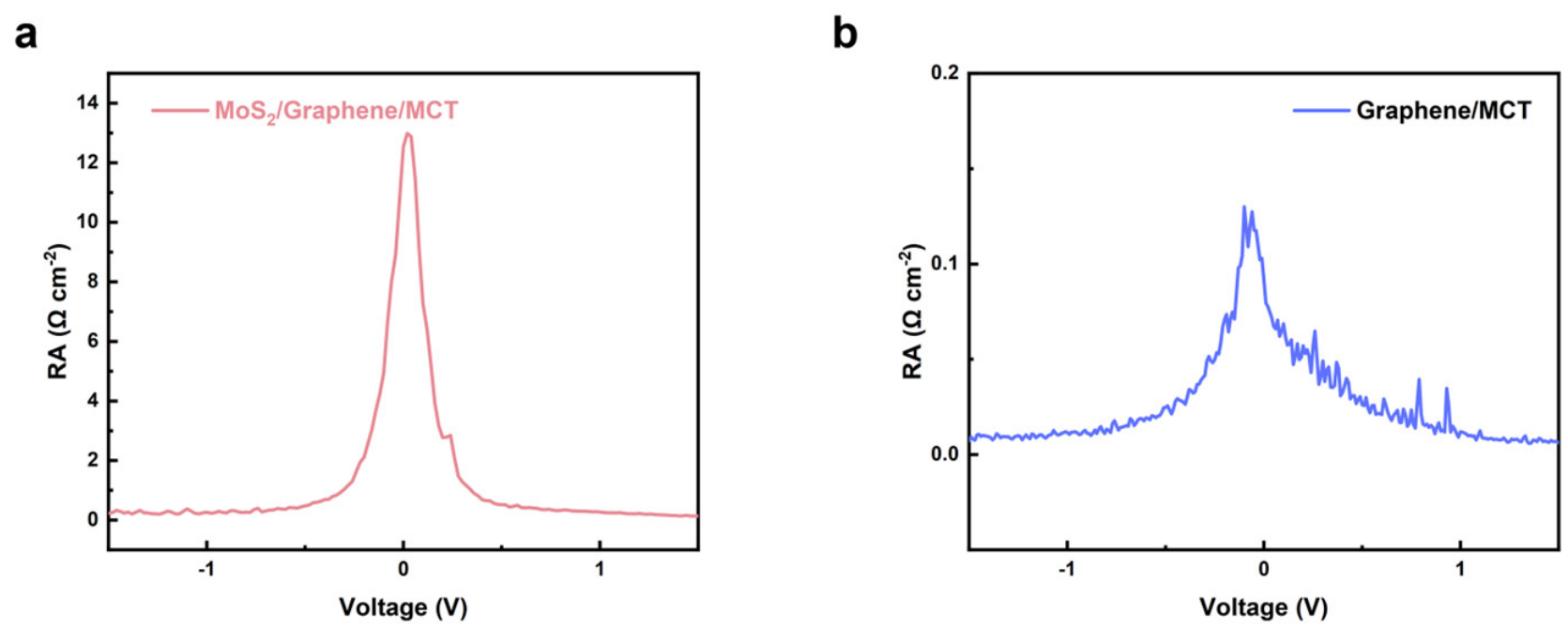

Fig. S18. Resistance-area product measurement of the **a** MoS_2/graphene/MCT vdW photodetector and **b** graphene/MCT heterojunction device.

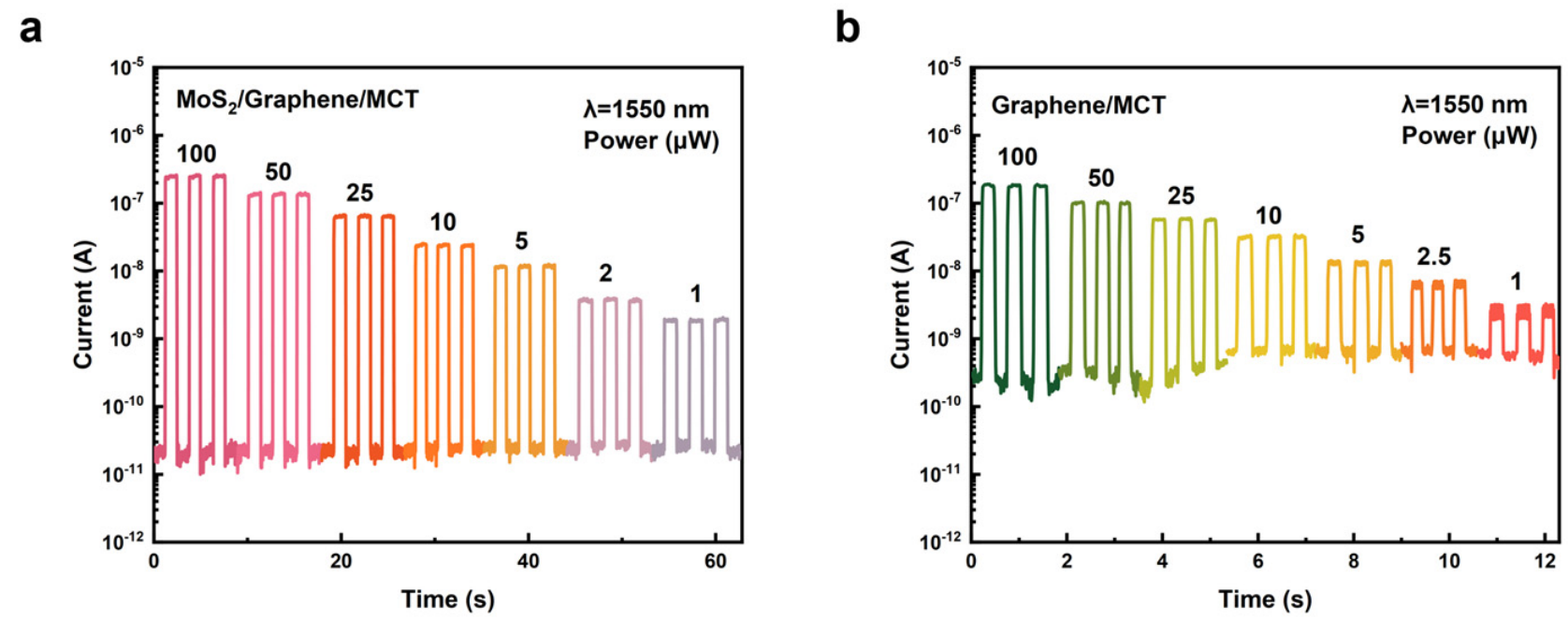

Fig. S19. Power-dependent photoresponse of the **a** MoS_2/graphene/MCT vdW photodetector and **b** graphene/MCT heterojunction device for 1550 nm in logarithmic scale.

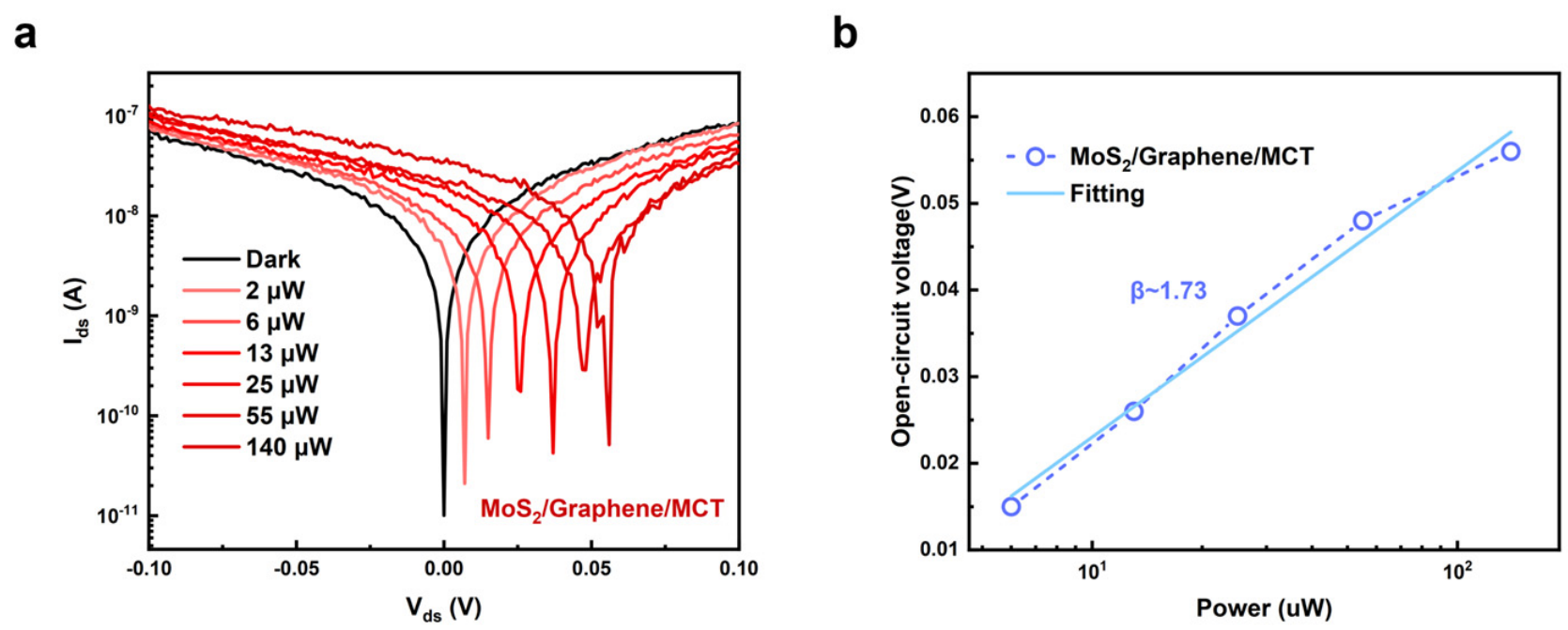

Fig. S20. The open-circuit characterization of the MoS_2/graphene/MCT vdW photodetector under 1550 nm focused laser. **a** Output characterization curves under various incident light powers of MoS_2/graphene/MCT vdW devices. **b** The incident light power dependence open-circuit voltage and fitting curve extracted from Fig. S20a.

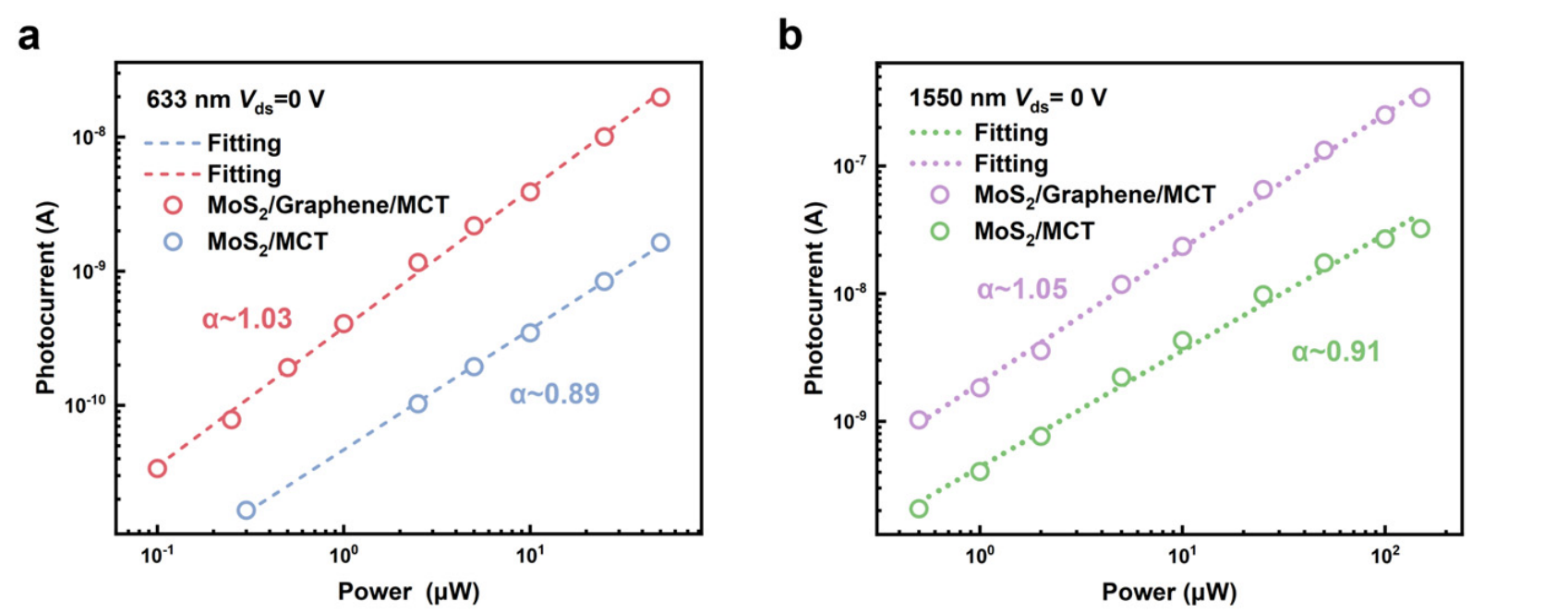

Fig. S21. Power-dependent photocurrent comparison between the MoS_2/MCT heterojunction and MoS_2/graphene/MCT vdW photodetector under **a** 633 nm and **b** 1550 nm.

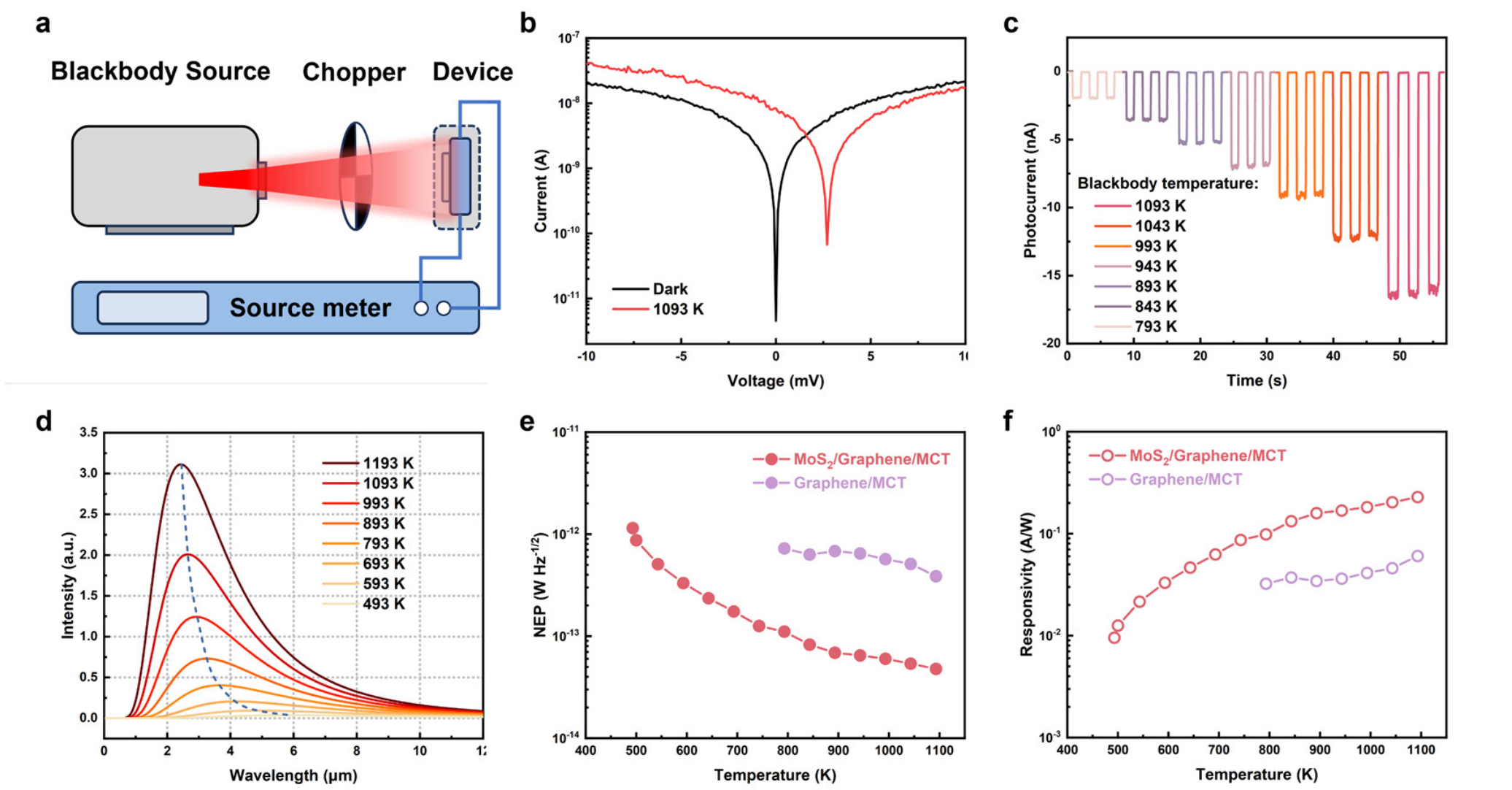

Fig. S22. Blackbody characterization of MoS_2/graphene/MCT vdW photodetector. **a** Schematic diagram of blackbody response measurement setup. **b** Output curves with and without 1093 K blackbody radiation illumination at room temperature. **c** Time-resolved photoresponse of MoS_2/graphene/MCT vdW photodetector under diverse blackbody temperature. **d** Spectra radiance versus wavelength from the 493 K to 1193 K blackbody temperatures. **e** Noise equivalent power (NEP) and **f** blackbody responsivity comparison between the graphene/MCT heterojunction and MoS_2/graphene/MCT vdW photodetector.

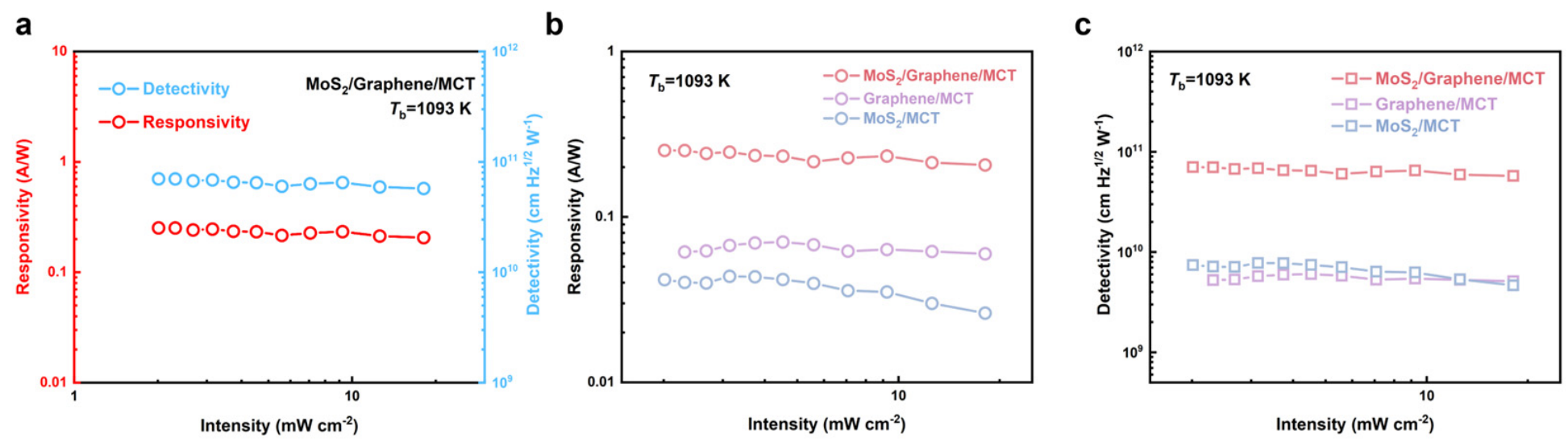

Fig. S23. Blackbody responsivity and detectivity characterization of the MoS_2/graphene/MCT vdW photodetector, graphene/MCT heterojunction, and MoS_2/MCT heterojunction. **a** Blackbody responsivity and detectivity of MoS_2/graphene/MCT vdW photodetector under diverse blackbody radiation intensity. **b** Blackbody responsivity and **c** detectivity comparison of graphene/MCT heterojunction, MoS_2/MCT heterojunction and MoS_2/graphene/MCT vdW photodetector under 1093 K blackbody temperature.

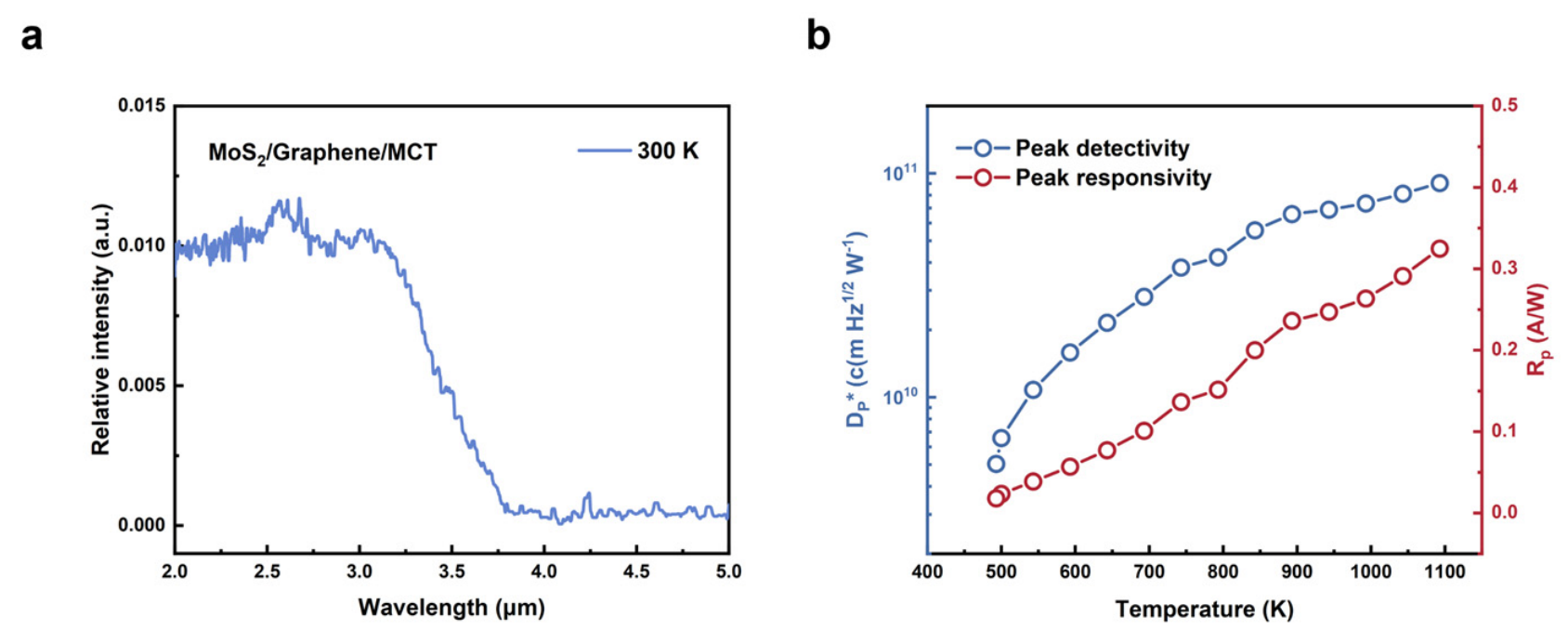

Fig. S24. Spectral response and blackbody detection performance of MoS_2/graphene/MCT vdW photodetector. **a** Relative response spectrum of the MoS_2/graphene/MCT vdW photodetector. **b** Peak photoresponsivity and detectivity under various blackbody temperatures radiation from 493 K to 1193 K.

Table S1. Performance comparison of MWIR photodetector[7-26]

Materials	Devices	λ_c (μm)	R_0A (Ω cm^{-2})	J_{dark} (A cm^{-2})	Responsivity (A W^{-1})	Response time	D^* (cm Hz$^{1/2}$W^{-1})	Reference
Traditional materials	HgCdTe (PV)	3.4	≥ 0.5	/	≥ 0.8	≤ 260 ns	7×10^9	Vigo
	HgCdTe (PV)	5.0	≥ 1	/	≥ 1	120 ns	1.5×10^{10}	Thorlabs
	InAs (PV)	3.5	0.75	/	0.9	35 ns	7×10^9	Vigo
	InAsSb (PV)	5.9	4	/	0.14	32 ns	2.3×10^9	Vigo
	InAsSb (pBin)	4.4	0.07	/	1.6	/	2.7×10^9	7
	T2SL InAs/GaSb	> 4.5	0.1	3×10^{-2}	0.2	/	4×10^8	8
	T2SL InAs/InAsSb	5.5	/	0.01	0.17	/	3×10^8	9
	T2SL InAs/GaSb	4.3	10	1.5×10^{-4}	0.15	/	3.7×10^9	10
	T2SL InAs/Al(In)Sb/GaAb	4.3	0.089	2×10^{-3}	0.13	/	9.7×10^8	11
Colloidal quantum dot	HgTe/Ag_2Te CQD	3.8	0.02	/	0.001	1 μs (80 K)	7.2×10^8	12
	HgTe CQD (PIN)	4.4	/	2.5×10^{-3}	0.58	<1 ms	7.6×10^9	13
	HgTe CQD (PN)	3.7	~0.2	/	/	/	1.2×10^9	14
2D materials	Graphene/MCT	4.0	/	1×10^{-4}	2.5	13 ns	2×10^{10}	15
	BP/MoS_2/BP	4.2	0.02 (1 mV)	4.6×10^{-2} (1 mV)	1.067	3.1 μs	8.61×10^{10}	16
	BP/Bi_2O_2Se	3.8	5	/	0.25 (Blackbody)	142 μs	1.14×10^{10} (1.3 μm laser)	17
	BP/MCT	4.4	/	1×10^{-4}	0.19	150 μs	7.9×10^{10}	18
	BP/MoS_2	4.0	2.75	/	0.9	3.7 μs	1.1×10^{10}	19
	Te/Graphene	3.5	/	2×10^{-5}	0.01	17 μs	3.7×10^8	20
	BP/MoS_2 with F-p Cavity	3	3.3	5×10^{-5}	0.1	< 3 ns	1.7×10^9	21
	BP/MoS_2/Si	3.9	0.92	3.5×10^{-3} (5 mV)	0.25	50 μs	6.4×10^9	22
	BP/MoS_2/B-AsP	4.9	/	7.7×10^{-4} (-0.1 V)	0.519	0.5 μs	6.2×10^9	23
	Graphene/metasurface	/	/	6.25 (0.1 V)	11.99 (0.1 V)	68 ms (0.1 V)	1×10^9 (0.1 V)	24
	PdSe/Graphene/Si	/	/	4.44×10^{-6}	/	100 μs	8.0×10^{10}	25
	Twist BP	4	/	/	0.05	100 μs	5×10^8	26
**This work**	**MoS_2/Graphene/MCT vdW heterostructure**	**3.6**	**13**	**2.8×10^{-6}**	**0.325**	**23 ns**	**8.0×10^{10}**	**This work**

R_0A: Resistance area product under zero bias; J_{dark}: Dark current density; CQD: Colloidal quantum dot; T2SL: Type-II superlattice.

Reference

1 Junhao Chu, *et al. Physics and Properties of Narrow Gap Semiconductors*. (2008).

2 C. R. Becker, V. *et al.* Band structure and its temperature dependence for type-III HgTe/$Hg_{1-x}Cd_xTe$ superlattices and their semimetal constituent. *PHYSICAL REVIEW B* **62** (2000).

3 Isaiah O Oladeji, L. *et al.* Metal/CdTe/CdS/Cd_{1-x}ZnxS/TCO/glass: A new CdTe thin film solar cell structure. *Solar Energy Materials and Solar Cells* **61**, 203-211 (2000).

4 H. Nishino, K. *et al.* Acceptor level related Shockley-Read-Hall centers in p-HgCdTe. *Journal of Crystal Growth*, 275-279 (2000).

5 Majkowycz, K. *et al.* New insight into defect energy levels in HgCdTe. *Infrared Physics & Technology* **137** (2024).

6 Chen, W. *et al.* Native point defects in HgCdTe infrared detector material: Identifying deep centers from first principles. *Journal of Applied Physics* **135**, 114502 (2024).

7 Xie, H. *et al.* Room-temperature InAsSb pBin detectors for mid-infrared application. *Infrared Physics & Technology* **128** (2023).

8 Serincan, U. *et al.* Direct growth of type II InAs/GaSb superlattice MWIR photodetector on GaAs substrate. *Superlattices and Microstructures* **120**, 15-21 (2018).

9 Huang, J. *et al.* High-Speed Mid-Wave infrared uni-traveling carrier photodetector based on InAs/InAsSb Type-II superlattice. *IEEE Electron Device Letters* **43**, 745-748 (2022).

10 Hackiewicz, K. *et al.* Influence of GaAs and GaSb substrates on detection parameters of InAs/GaSb superlattice-based mid-infrared interband cascade photodetectors. *Appl Opt* **59**, E42-E47 (2020).

11 Johnson, M. B. *et al.* Midwavelength interband cascade infrared photodetectors with superlattice absorbers and gain. *Optical Engineering* **57** (2017).

12 Ackerman, M. M. *et al.* Fast and sensitive colloidal quantum dot mid-wave

infrared photodetectors. *ACS Nano* **12**, 7264-7271 (2018).

13 Xue, X. *et al.* High-operating-temperature mid-infrared photodetectors via quantum dot gradient homojunction. *Light Sci Appl* **12**, 2 (2023).

14 Peterson, J. *et al.* Room-temperature 15% efficient mid-infrared HgTe colloidal quantum dot photodiodes. *ACS Appl Mater Interfaces* **15**, 19163-19169 (2023).

15 Wang, Y. *et al.* Fast uncooled mid-wavelength infrared photodetectors with heterostructures of van der Waals on epitaxial HgCdTe. *Advanced Materials* **34** (2021).

16 Wang, F. *et al.* Fully depleted self-aligned heterosandwiched van der Waals photodetectors. *Advanced Materials* **34** (2022).

17 Chen. Y *et al.* Momentum-matching and band-alignment van der Waals heterostructures for high-efficiency infrared photodetection. *Science Advances* **8** (2022).

18 Jiao, H. *et al.* HgCdTe/Black Phosphorus van der Waals heterojunction for high-performance polarization-sensitive midwave infrared photodetector. *Science Advances* **8** (2022).

19 Bullock, J. *et al.* Polarization-resolved black phosphorus/molybdenum disulfide mid-wave infrared photodiodes with high detectivity at room temperature. *Nature Photonics* **12**, 601-607 (2018).

20 Peng, M. *et al.* Room-temperature blackbody-sensitive and fast infrared photodetectors based on 2D tellurium/graphene van der Waals heterojunction. *ACS Photonics* **9**, 1775-1782 (2022).

21 Yan, W. *et al.* Spectrally selective mid-wave infrared detection using fabry-pérot cavity enhanced black phosphorus 2D photodiodes. *ACS Nano* **14**, 13645-13651 (2020).

22 Wu, P. *et al.* Van der Waals two-color infrared photodetector. *Light: Science & Applications* **11** (2022).

23 Zhang, S. *et al.* Multi-dimensional optical information acquisition based on a misaligned unipolar barrier photodetector. *Nat Commun* **15**, 7071 (2024).

24 Jiang, H. *et al.* Synergistic-potential engineering enables high-efficiency

graphene photodetectors for near- to mid-infrared light. *Nat Commun* **15**, 1225 (2024).

25 Yang, C. *et al.* PbSe-modified graphene/Si heterojunction for self-powered broadband detection with dual-Light modulation Effects. *ACS Appl Mater Interfaces* **17**, 56299-56307 (2025).

26 Jiang, H. *et al.* Twist-stacked black phosphorus for wide-spectral chiral photodetection. *Nat Commun* (2026).
